# Risk factors and aetiological agents of community-acquired pneumonia in children under five years in South Asia: A systematic review

**DOI:** 10.1371/journal.pgph.0006388

**Published:** 2026-11-10

**Authors:** Sumaya Ferdowsi, Md Mamunur Rashid Sheikh

**Affiliations:** 1 Paediatric Neurology and Development, Sir Salimullah Medical College Mitford Hospital, Dhaka, Bangladesh; 2 Department of Social Work, Jagannath University, Dhaka, Bangladesh; Directorate of Factories, INDIA

## Abstract

Pneumonia remains a leading cause of mortality among children under five years in South Asia, a region that, with sub-Saharan Africa, accounts for over half of the world's childhood pneumonia cases. To our knowledge, no prior review has jointly synthesised the risk factors and aetiological agents of community-acquired pneumonia (CAP) in under-fives specific to this region. This systematic review aimed to identify, critically appraise, and synthesise available evidence on the risk factors and aetiological agents associated with CAP in children under five years of age in South Asian countries. Following PRISMA guidelines, four electronic databases (MEDLINE, PubMed, Global Health, and Web of Science) were systematically searched for studies published between January 2000 and July 2024, with the search updated to January 2026. Quality assessment was performed using the Crowe Critical Appraisal Tool (CCAT), with only high-quality studies (≥75% score) included in the final synthesis. Data were synthesised narratively due to heterogeneity in study designs and outcome measures. From 1196 records identified and 747 studies screened, 16 met the inclusion criteria after quality assessment (8 on risk factors, 8 on aetiology), conducted across five South Asian countries (India, Bangladesh, Nepal, Pakistan, Bhutan). Young age (children 0–23 months), malnutrition (particularly underweight and wasting), and incomplete immunisation emerged as the most consistently identified risk factors across studies. Environmental exposures, including indoor air pollution, poor ventilation, and overcrowding, were significant contributors. Viral pathogens predominated in aetiological studies, with respiratory syncytial virus being the most frequently identified pathogen (mean detection rate 29.2%). Blood culture positivity for bacterial pathogens was generally lower (1.5-31.3%; below 13% in four of five studies), with *Streptococcus pneumoniae* and *Staphylococcus aureus* most common among positive cultures. This systematic review demonstrates that childhood CAP in South Asia results from a complex interplay of modifiable host, environmental, and socioeconomic risk factors. Integrated interventions addressing nutrition, immunization coverage, household air pollution, and socioeconomic determinants are essential for reducing the substantial pneumonia burden among South Asian children.

## Introduction

Pneumonia remains one of the most significant causes of morbidity and mortality among children under five years of age worldwide, contributing to approximately 14% of all under-five deaths globally [1]. The United Nations Millennium Development Goal 4 (MDG 4) targeted a two-thirds reduction in under-five mortality between 1990 and 2015, achieving a 53% reduction by the end of this period [2]. Subsequently, global under-five mortality continued to decline, with a 60% overall reduction observed between 1990 and 2022, decreasing from 93 to 37 deaths per 1,000 live births [3]. Despite this substantial progress, child survival remains a critical public health priority, with approximately 13,400 preventable under-five deaths occurring daily in 2022 [3].

The global burden of childhood pneumonia is disproportionately concentrated in low- and middle-income countries (LMICs), particularly in South Asia and sub-Saharan Africa [4,5]. A systematic review estimated that the incidence of pneumonia in developing countries is approximately 0.22 episodes per child per year [6]. Among the estimated 156 million new paediatric pneumonia cases occurring globally each year, approximately 61 million occur in the Southeast Asia and Pacific region alone [7]. South Asian countries bear an exceptionally high burden: India, Bangladesh, and Pakistan are among a group of five countries that collectively account for more than half of the world's new cases of clinical pneumonia [8]. The proportion of under-five deaths attributable to pneumonia varies considerably across the region, ranging from 6% in Sri Lanka to 23% in Afghanistan [8].

Community-acquired pneumonia (CAP) refers to pneumonia with signs and symptoms acquired outside of hospital settings, distinguishing it from nosocomial or healthcare-associated pneumonia [9]. Lower respiratory tract infections, including pneumonia, bronchitis, and bronchiolitis, represent a spectrum of respiratory illness, with pneumonia being characterised by infection of the lung parenchyma [10]. The World Health Organization (WHO) case definitions for childhood pneumonia are widely used in clinical and research settings, defining pneumonia based on the presence of cough or difficulty breathing with age-specific respiratory rate thresholds: more than 60 breaths per minute for infants under two months, more than 50 breaths per minute for children aged 2–11 months, and more than 40 breaths per minute for children aged 12–59 months [11]. Severe pneumonia is characterised by additional danger signs, including oxygen saturation below 90%, central cyanosis, grunting, severe chest indrawing, or general danger signs such as inability to breastfeed, lethargy, altered consciousness, or convulsions [11].

Notably, while overall under-five mortality has declined substantially over recent decades, the reduction in pneumonia-related mortality has been slower compared to other major causes of childhood death. Between 2000 and 2015, pneumonia-related deaths decreased by 46%, compared to a 57% reduction in diarrhoea-related deaths over the same period [12]. This differential progress highlights pneumonia as a persistent and challenging public health problem requiring renewed attention and targeted interventions.

The aetiology of childhood pneumonia has undergone a significant epidemiological transition over recent decades, particularly following the widespread implementation of conjugate vaccination programmes against *Haemophilus influenzae* type b (Hib) and *Streptococcus pneumoniae* [13]. In developed countries with high vaccination coverage and improved sanitation and hygiene, viral pathogens have emerged as the predominant causes of childhood pneumonia: a large U.S. cohort study found viruses in 66% of hospitalised children with community-acquired pneumonia compared with bacteria in only 8%, replacing the historical bacterial dominance [14]. The landmark Pneumonia Aetiology Research for Child Health (PERCH) study, conducted across multiple sites in Africa and Asia, demonstrated that in the post-conjugate vaccine era, viruses accounted for approximately 61% of pneumonia cases, while bacteria accounted for 27% [15]. Respiratory syncytial virus (RSV) emerged as the single most important pathogen, accounting for 31% of cases, followed by other respiratory viruses, including human metapneumovirus, parainfluenza viruses, and influenza viruses [15]. Among bacterial pathogens, *S. pneumoniae* and *Staphylococcus aureus* were most frequently implicated in severe pneumonia cases [15].

The risk factors for childhood pneumonia are multifactorial, involving complex interactions between host susceptibility, environmental exposures, and infectious agents. Host-related factors include nutritional status, immunisation status, age, gender, and presence of comorbid conditions [16]. Malnutrition, particularly undernutrition, compromises immune function and has been consistently associated with increased pneumonia susceptibility and severity [17,18]. Lack of exclusive breastfeeding during the first six months of life has been shown to increase the risk of CAP by 1.5-2.6 times and contributes to a 30–42% increase in respiratory infection incidence in developing countries [16]. Iron deficiency anaemia represents another important nutritional risk factor, with some studies demonstrating a five to seven-fold increase in pneumonia risk among anaemic children [16].

Environmental factors play a crucial role in childhood pneumonia susceptibility in LMICs. Household air pollution from biomass fuel combustion for cooking and heating represents a major environmental risk factor, with multiple studies demonstrating odds ratios ranging from 2 to 4 for acute lower respiratory infections (ALRI) in children exposed to biomass smoke [19–22]. Parental smoking, particularly maternal smoking, contributes additional respiratory health risks through second-hand smoke exposure [16]. Overcrowding and poor housing ventilation facilitate respiratory pathogen transmission and have been associated with increased pneumonia risk in multiple settings [23]. Socioeconomic factors, including low parental education, household poverty, and poor housing quality, operate through multiple pathways to influence pneumonia risk, affecting nutritional status, healthcare access, immunisation uptake, and environmental exposures [16,24].

Understanding the specific epidemiology of childhood pneumonia in South Asian populations is essential for several reasons. First, the region bears a disproportionate share of the global pneumonia burden, yet regional data are often underrepresented in global estimates. Second, risk factor profiles may differ from those observed in other settings due to unique cultural, environmental, and socioeconomic contexts. Third, vaccination coverage and programme implementation vary across South Asian countries, potentially influencing aetiological patterns. Fourth, accurate local data on disease burden, risk factors, and pathogens are essential for developing context-appropriate prevention strategies, treatment guidelines, and vaccination priorities [25].

Several systematic reviews have examined childhood pneumonia risk factors and aetiology in various global contexts. A systematic review by von Mollendorf et al. [26] examined pneumonia aetiology across all LMICs, while Soelaeman and Cahyadirga [24] reviewed risk factors in selected Asian countries. These reviews differ from the present work in both scope and focus. Von Mollendorf et al. [26] synthesised aetiological data across all low- and middle-income countries, so South Asian findings were pooled with those from other regions and were not disaggregated, and risk factors were outside the review's remit. Soelaeman and Cahyadirga [24] addressed risk factors in selected Asian countries but did not examine aetiological agents, and their geographic coverage extended beyond South Asia. Broader reviews of childhood pneumonia in developing countries, such as those by Rudan et al. and DeAntonio et al., have likewise reported risk factors and pathogens at a global or multi-region level and were not restricted to South Asia or to community-acquired pneumonia in children under five. Consequently, no existing review brings both bodies of evidence together for the South Asian under-five population, or appraises study quality across them, leaving clinicians and policymakers in the region without a single synthesis linking who is at risk to which pathogens are responsible. However, to best of our knowledge, no systematic review has comprehensively examined both risk factors and aetiological agents of CAP specifically in South Asian children under five years of age. Given the substantial pneumonia burden in this region and the need for targeted interventions, a focused systematic review synthesising available evidence from South Asian countries is warranted.

The objectives of this systematic review are: (1) to identify the aetiological agents associated with CAP in children under five years in South Asian countries; and (2) to explore the potential risk factors associated with CAP development in children under five years in South Asian countries. The findings will inform evidence-based recommendations for pneumonia prevention strategies, treatment guidelines, and research priorities in the region.

## Methods

### Protocol and registration

This systematic review was conducted following the Preferred Reporting Items for Systematic Reviews and Meta-Analyses (PRISMA) guidelines to ensure comprehensive and transparent reporting [27]. At the time of initiation, the review protocol was not prospectively registered in platforms such as PROSPERO or the Open Science Framework. This was because the review was conducted as part of a time-limited academic dissertation, and registration was not a requirement of the degree programme. We acknowledge this as a limitation. The review methodology was designed using the PICO framework: Population (children under five years living in South Asian countries), Intervention (not applicable), Comparison (no comparison groups), and Outcomes (risk factors and aetiological agents associated with CAP).

### Eligibility criteria

Studies were included if they met all of the following criteria: (1) examined risk factors and/or aetiological agents of community-acquired pneumonia; (2) included children under five years of age (0–59 months); (3) were conducted in South Asian countries, defined as India, Bangladesh, Pakistan, Nepal, Bhutan, Sri Lanka, Afghanistan, or Maldives; (4) were published in English; Language was restricted to English because no translation resources were available to the review team; this restriction is acknowledged as a potential source of language bias and is discussed under Limitations (5) employed observational study designs including cross-sectional studies, case-control studies, cohort studies, or case series; (6) were published as full-text peer-reviewed journal articles; and (7) were published between January 2000 and July 2024, with the database search subsequently updated in 2026 to capture studies published after this date. Multi-country or multi-regional studies were included if South Asian data could be extracted separately.

Studies were excluded if they: (1) focused on other respiratory conditions such as bronchiolitis, bronchitis, or asthma without separate pneumonia data; (2) reported on adult pneumonia or mixed adult-paediatric populations without separate under-five data; (3) lacked information about the country setting; (4) were not full-text, peer-reviewed primary journal articles (including conference abstracts and posters, newspaper articles, guidelines, and book chapters). We acknowledge that some book chapters and conference proceedings undergo peer review; these publication types were nonetheless excluded because they typically do not report the full methodological detail (sampling, case definitions, analysis, and effect estimates with confidence intervals) required for data extraction and for quality appraisal with the CCAT, and because their peer-review status frequently cannot be verified reliably. No source identified in the search was excluded on this basis alone that reported extractable primary data meeting all other criteria; or (5) examined only pneumonia mortality or treatment outcomes without reporting risk factors or aetiology.

### Information sources and search strategy

A comprehensive literature search was conducted across four electronic databases accessed through the EBSCOhost platform: MEDLINE Complete, PubMed, Global Health, and Web of Science. The search was performed between June and July 2024. An updated search using the same strategy and the same four databases was conducted in January 2026, covering studies published from August 2024 to January 2026. Additionally, reference lists of included studies and relevant systematic reviews were manually searched to identify potentially eligible studies not captured in the electronic database search.

The search strategy was developed in consultation with information specialists and employed a combination of free-text keywords and Medical Subject Headings (MeSH) terms. Search terms were organized into four conceptual categories combined with Boolean operators: (1) Outcome terms: (aetiology OR etiology OR “causative organism*” OR “risk factor*” OR predictor*); (2) Condition terms: (“community acquired pneumonia” OR pneumonia OR “lower respiratory tract infection*”); (3) Population terms: (“children under five*” OR children OR infant* OR paediatric* OR pediatric* OR “young infant*” OR neonate*); and (4) Geographic terms: (“South Asia*” OR India OR Bangladesh OR Pakistan OR Nepal OR Bhutan OR “Sri Lanka” OR Afghanistan OR Maldives). MeSH terms including “Risk Factors,” “Pneumonia,” “Infant,” “Child, Preschool,” and “Asia, Southern” were incorporated where applicable. The complete search strategy for each database is provided in S1 Text.

### Study selection process

All records identified through database searching were imported into EndNote reference management software. Duplicate records were identified and removed using EndNote's duplicate detection function, supplemented by manual review. The remaining records underwent two-stage screening. In the first stage, titles and abstracts were screened against predefined eligibility criteria by the primary reviewer. Studies clearly not meeting the inclusion criteria were excluded at this stage, with reasons documented. In the second stage, full-text articles of potentially eligible studies were retrieved and assessed for eligibility by the primary reviewer. To enhance reliability, a random 10% sample of records was independently screened by a second reviewer, and discrepancies were resolved through discussion. The second reviewer was independent of the review team and not involved in study conduct; screening was performed independently and blinded to the primary reviewer's decisions, and any disagreements were resolved by discussion until consensus was reached, with no third reviewer required. The complete study selection process was documented using a PRISMA flow diagram that recorded the number of studies at each stage and the reasons for exclusion (Fig 1).

**Fig 1:**
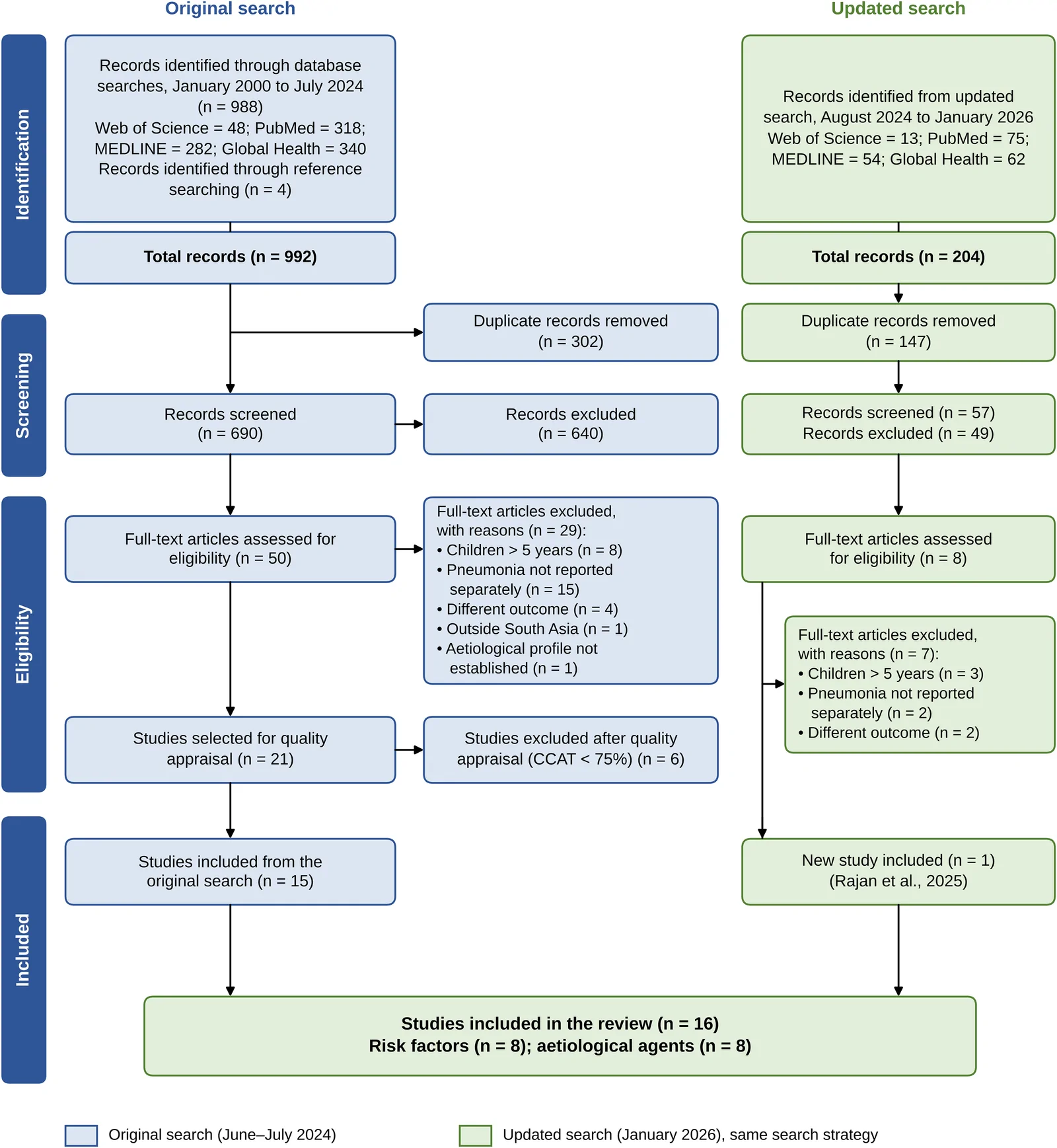
PRISMA flow diagram of study selection. Records identified through the original search (June–July 2024) across Web of Science, PubMed, MEDLINE and Global Health.

### Data extraction

Data were extracted from included studies using a standardised data extraction form developed for this review. The following information was extracted: study identification (author, year of publication, journal name), study characteristics (country, study design, study setting, study period), population characteristics (sample size, age range of participants, pneumonia definition used), risk factors examined and reported associations (odds ratios, confidence intervals, p-values), aetiological agents identified (pathogens detected, diagnostic methods used, specimen types, detection rates), and key findings and conclusions. For studies reporting on risk factors, both statistically significant and non-significant associations were extracted to provide a comprehensive picture of the evidence (details of the characteristics of the included studies are found in S2 Table and S3 Table

### Quality assessment

The methodological quality of included studies was assessed using the Crowe Critical Appraisal Tool (CCAT) [28,29]. The CCAT is a validated instrument applicable to various quantitative and qualitative research designs, enabling consistent evaluation across different study types. The tool assesses eight categories: preliminaries, introduction, design, sampling, data collection, ethical matters, results, and discussion. Each category is scored from 0 to 5, yielding a maximum possible score of 40 points. The scoring system classifies sources as: 4–5 (high quality within category), 2–3 (moderate quality), and 0–1 (low quality) [28].

Overall study quality was classified based on the percentage of maximum possible score achieved: high quality (75–100%), acceptable quality (51–74%), and poor quality (below 50%) [30]. Studies classified as poor quality (scoring below 75%) were excluded from the final synthesis to ensure that conclusions were based on methodologically robust evidence. Individual category scores were also examined to identify specific methodological strengths and weaknesses across included studies (see S1 Table).

### Data synthesis and analysis

Due to substantial heterogeneity in study designs, populations, outcome definitions, measurement methods, and reporting formats across included studies, statistical meta-analysis was not appropriate. Instead, a narrative synthesis approach was employed to systematically organise, describe, and interpret the findings. Risk factors were categorised into three domains: host-related factors, environment-related factors, and socioeconomic factors. Aetiological agents were categorised as viral pathogens or bacterial pathogens. Within each category, findings were synthesised narratively, with attention to consistency of findings across studies, strength of associations reported, and potential sources of heterogeneity. Study characteristics and findings are presented in summary tables to facilitate comparison and interpretation.

## Results

### Study selection

The systematic database search identified 988 records: Web of Science (n = 48), PubMed (n = 318), MEDLINE (n = 282), and Global Health (n = 340). An additional 4 records were identified through manual searching of reference lists, yielding a total of 992 records. Following duplicate removal (n = 302), 690 unique records underwent title and abstract screening. Of these, 640 records were excluded for not meeting the inclusion criteria based on title and abstract review. The remaining 50 articles were retrieved for full-text assessment.

At the full-text stage, 29 articles were excluded for the following reasons: 15 studies reported on acute respiratory infections (ARI) or acute lower respiratory infections (ALRI) generally without separate pneumonia-specific data; 8 studies included children over five years of age without separate under-five data; 4 studies focused on different outcomes (pneumonia mortality or treatment outcomes rather than risk factors or aetiology); and 1 study was conducted outside South Asia. One additional study was excluded because the aetiological profile was not adequately established. The remaining 21 studies proceeded to quality assessment. Quality assessment using the CCAT resulted in the exclusion of 6 studies scoring below 75% (classified as poor quality, range 42.5-73%). The final synthesis included 15 studies: 8 examining risk factors and 7 examining aetiological agents. The study selection process is illustrated in the PRISMA flow diagram (Fig 1). The search was subsequently updated using the same strategy across all four databases to cover the period August 2024 to January 2026. This update identified 204 records (Web of Science n = 13, PubMed n = 75, MEDLINE n = 54, Global Health n = 62). Following duplicate removal (n = 147), 57 records were screened and 49 were excluded at title and abstract. Of the 8 articles retrieved for full-text assessment, 7 were excluded (3 included children over five years of age without separate under-five data; 2 did not report pneumonia separately; 2 focused on different outcomes). The remaining study (Rajan et al., 2025) met all inclusion criteria and was retained following CCAT appraisal (32/40, 80%), increasing the final number of included studies to 16 (8 examining risk factors and 8 examining aetiological agents). Across the original and updated searches, 1,196 records were identified and 747 unique records were screened.

### Study characteristics

The 16 included studies were conducted across five South Asian countries: India (n = 4), Bangladesh (n = 4), Nepal (n = 4), Pakistan (n = 3), and Bhutan (n = 1). Study designs varied and included case-control studies (n = 8, representing 44% of included studies), cross-sectional studies (n = 3), prospective cohort studies (n = 3), secondary analysis of survey data (n = 1), and active surveillance (n = 1). Sample sizes ranged considerably, from 66 participants in the smallest study to 7,026 in the largest multicentric cohort study. All studies focused on children under five years of age, with most using age ranges of 2–59 months or 0–59 months, consistent with WHO age classifications for childhood pneumonia.

Among the 8 studies examining risk factors, 5 employed case-control designs, comparing pneumonia cases with controls without pneumonia or with upper respiratory infections only. One study used cross-sectional designs, one conducted a secondary analysis of national survey data, and one was a prospective cohort study. Most risk factor studies examined multiple potential risk factors simultaneously, while three focused on specific risk factors: one on household air quality, one on arsenic exposure, and one on predictors of recurrent pneumonia.

The 8 studies examining aetiology employed various diagnostic approaches. Two studies used cross-sectional designs, three used case-control designs, and two were a prospective cohort study. Specimen types included blood cultures (in 5 studies), nasopharyngeal aspirates or swabs (8 studies), induced sputum (1 study), and pleural fluid (1 study), and tracheal aspirates and bronchoalveolar lavage (1 study). Diagnostic methods included bacterial culture, enzyme-linked immunosorbent assay (ELISA), and molecular methods, including reverse transcriptase polymerase chain reaction (RT-PCR) and multiplex PCR. Four studies examined both bacterial and viral pathogens, three focused primarily on viral agents, and one examined bacterial pathogens only.

The definition of pneumonia varied across studies, though most used WHO criteria or modified WHO criteria. Some studies used clinical criteria alone, while others required radiological confirmation. This variation in case definitions represents an important source of heterogeneity across studies. Detailed characteristics of all included studies are presented in S2 Table and S3 Table.

### Quality assessment results

Quality assessment scores for the 22 studies undergoing appraisal ranged from 17 to 39 out of a maximum possible 40 points (42.5% to 97.5%). The highest-scoring study, a prospective cohort study from Bhutan by Jullien et al. [25], achieved 39 points (97.5%), while the lowest-scoring study achieved only 17 points (42.5%) and was excluded from the synthesis. Six studies scoring below 75% (range 42.5-72.5%) were excluded. Among the 16 studies included in the final synthesis, all scores 75% or above: three achieved 91% or higher (93–98%) and thirteen achieved scores between 75–90%. The additional study identified through updated search (Rajan et al., 2025) was appraised using the same CCAT instrument and scored 80%, placing it in the high quality band; its eight category scores are presented in S1 Table.

Examination of individual CCAT categories revealed specific patterns in methodological quality. Study design quality was generally high, with 15 studies achieving scores of 4 or 5 out of 5 in this category. Data collection methods showed more variability, with scores ranging from 2 to 5 and 17 studies achieving high scores. Sampling methods represented an area of relative weakness, with only 14% of studies achieving the highest score of 5, and 2 studies receiving the lowest scores. Ethical considerations showed the greatest variability, with scores ranging from 0 to 5; notably, 19% of studies received scores of 0 or 1, indicating inadequate reporting or attention to ethical matters, while 14% received scores of 5. Results reporting was generally strong, with 29% of studies achieving scores of 5 in this category. The detailed quality assessment scores for each included study are presented in S1 Table.

### Risk factors for community-acquired pneumonia

Eight studies meeting quality criteria examined risk factors for childhood CAP across four South Asian countries: India (n = 2), Bangladesh (n = 2), Nepal (n = 2), and Pakistan (n = 2). These studies collectively identified 16 statistically significant risk factors, which were categorised into three domains: host-related, environment-related, and socioeconomic. The findings from each study are summarised in Table 1.

**Table 1:** Findings of included studies (studies on risk factors).

	Author & Year of Publication	Country	Age and Number of Participants	Risk factors		
				Statistically significant factors	Not significant factors	Not associated factors
1	Ram et al. (2014)	Bangladesh	Under five years. 331 cases,663 controls	1. Overcrowding 2. Lack of cross ventilation 3. Gender: male child 4. Living space covered with a tin roof Poor socioeconomic status.	1. High level of particulate matter in indoor air	
2	George et al. (2015)	Bangladesh	28 days -59 months. 153 cases,296 controls	Arsenic exposure		
3	Gothankar et al. (2018)	India	Under-five. 3671	1.Partial immunisation (OR=2.41,95% CI;1.06-5.49) 2. Mothers awareness about air pollution and overcrowding (OR=0.68,95%CI;0.52-0.88; odds higher among aware mothers - reverse association) 3. Mothers awareness about symptoms of pneumonia (OR=0.36,95%CI;0.26-0.48; odds higher among aware mothers - reverse association)		1. Overcrowding 2. Inadequate ventilation 3. Type of fuel used(p>0.05) 4. Exclusive breastfeeding (p>0.05) 5. Wasting(p>0.05)
4	Goyal et al. (2021)	India	2-59 months. 7026	1. Younger children 2. Undernutrition 3. Hib vaccination (higher odds; AOR 1.81, 95% CI 1.53-2.13)	1. Male gender 2. Influenza and pneumococcal vaccination	
5	Karki et al. (2014)	Nepal	1month-5 years. 50 cases,150 controls	1. Children older than three years 2. Diarrhoea in the past three months (protective factor) 3. Chulo/stove produces smoke	1. Maternal age 2. Maternal education 3. Overcrowding 4. Low birth weight 5. Gestational age at birth 6. Colostrum feeding 7. Partial Immunisation 8. Poor ventilation 9. Parents smoking 10. Child attending a childcare centre (significant only before adjustment; AOR 1.66, 95% CI 0.70-3.97)	1. Exclusive breastfeeding
6	Dharel et al. (2022)	Nepal	0-59 months. 6658	1. Children aged 0-23 months had 1.5times the odds compared to 24-59 months 2. Children living in rural areas 3. Underweight 4. Currently smoking mothers 5. Non-disadvantaged ethnicity/caste	1. Maternal education 2. Wealth quintile 3. Location of kitchen and fuel used	
7	Fatmi & White (2002)	Pakistan	Under five years. 259 cases,187 controls	1. Younger age 2. Wasting 3. Children born at home 4. H/O neonatal chest illness 5. Previous h/o pneumonia 6. Lack of immunisation	1. Gender 2. Education of parents 3. Occupation of fathers 4. Underweight and stunting 5. H/O previous measles 6. Delayed initiation of breastfeeding	1. Number of siblings
8	Brown et al. (2020)	Pakistan	2-60 months	1. Poor housing quality 2. Incomplete vaccination (inverse association: lower odds of recurrence; matched AOR 0.61, 95% CI 0.40-0.94) 3. Smoker in household (inverse association; matched AOR 0.53, 95% CI 0.30-0.91)		1. Undernutrition 2. Sex of child 3. Inadequate ventilation 4. Absence of Breastfeeding 5. Type of household fuel used 6. Sibling number 7. Crowding 8. Household pet animals 9. Sanitation 10. Traffic fumes

#### Host-related risk factors.

Age of the child emerged (Table 1) as the most consistently identified host-related risk factor, with four studies demonstrating significantly higher pneumonia risk in younger children compared to older children within the under-five age group [31–33]. Dharel et al. [32] reported that children aged 0–23 months had 1.5 times higher odds of developing pneumonia compared to children aged 24–59 months (adjusted odds ratio [AOR] = 1.5, 95% confidence interval [CI]: 1.0-2.3). Goyal et al. [31] similarly found younger age to be an independent risk factor for pneumonia after multivariate adjustment (AOR 0.97 per month of age, 95% CI 0.97-0.98), as did Fatmi and White [33], although the confidence interval of their estimate included unity (AOR 1.01 per preceding month, 95% CI 0.99-1.03). Interestingly, one study by Karki et al. [34] from Nepal reported contrasting findings, with children older than three years showing a higher risk of hospital admission with pneumonia, though this may reflect healthcare-seeking patterns rather than true incidence differences.

Malnutrition was identified as a significant risk factor in three studies, though the specific anthropometric indicators associated with pneumonia varied. Dharel et al. [32] found that underweight children had 2.3 times higher odds of pneumonia compared to children with normal weight-for-age (AOR = 2.3, 95% CI: 1.4-3.9). Fatmi and White [33] reported that wasting (low weight-for-height) was significantly associated with pneumonia but found no significant association with underweight (low weight-for-age) or stunting (low height-for-age). Goyal et al. [31] similarly identified undernutrition as an independent risk factor. One study by Gothankar et al. [35] from India did not find statistically significant associations between wasting and pneumonia, highlighting some inconsistency in findings across studies.

Immunisation status was significantly associated with pneumonia risk in two studies. Gothankar et al. [35] found that partially immunised children had 2.4 times higher odds of pneumonia compared to fully immunised children (OR = 2.41, 95% CI: 1.06-5.49). Fatmi and White [33] reported that incomplete or absent immunisation was associated with increased pneumonia risk in their cohort. In contrast, Brown et al. [36] found that children without age-adequate vaccination had lower odds of recurrent pneumonia (matched AOR 0.61, 95% CI 0.40-0.94), which the authors attributed to confounding; Goyal et al. [31] reported higher odds of pneumonia among Hib-vaccinated children (AOR 1.81, 95% CI 1.53-2.13); and Karki et al. [34] found no significant association. These findings suggest that the protective effect of vaccination may be context-dependent or modified by other factors such as vaccine types included and coverage levels achieved.

Other host-related factors identified in individual studies included male gender, which was associated with increased pneumonia risk in one study [37], though several other studies found no significant gender differences. Health-related conditions and comorbidities were examined in two studies. Fatmi and White [33] found that previous history of pneumonia (AOR = 1.77, 95% CI: 1.16-2.7) and history of chest illness in the neonatal period (OR = 2.15, 95% CI: 1.20-3.87) were significant risk factors. Interestingly, Karki et al. [34] found that having diarrhoea within the previous three months was protective against pneumonia, possibly reflecting the effects of zinc supplementation or antibiotic treatment received during diarrhoea episodes. Home delivery was associated with increased pneumonia risk in one Pakistani study [33], possibly reflecting limited access to early neonatal care and immunisations.

#### Environment-related risk factors.

Indoor smoke exposure emerged as the most important environment-related risk factor, identified in multiple studies through different exposure measurements. Karki et al. [34] found that children living in houses with indoor smoke-producing stoves (chulo) had nearly four times higher odds of developing pneumonia (OR = 3.76, 95% CI: 1.20-11.82). Dharel et al. [32] reported that children of currently smoking mothers had 2.5 times higher odds of pneumonia (AOR = 2.5, 95% CI: 1.1-5.7). The mechanism likely involves damage to the respiratory epithelium, impairment of mucociliary clearance, and alteration of immune responses to respiratory pathogens [38,39].

Household ventilation and overcrowding were examined in several studies with mixed findings. Ram et al. [37] from Bangladesh found that adequate cross-ventilation of living spaces was protective against pneumonia (AOR = 0.72, 95% CI: 0.53-0.98), and overcrowding was associated with increased risk. However, Gothankar et al. [35] from India did not find statistically significant associations between overcrowding, inadequate ventilation, or cooking fuel type and pneumonia occurrence. These inconsistent findings may reflect differences in how these exposures were measured across studies or genuine contextual differences between urban Bangladesh and rural India study settings.

One study identified a unique environmental risk factor specific to the Bangladesh context. George et al. [40] found that children with elevated creatinine-adjusted urinary arsenic concentrations had approximately double the odds of developing pneumonia compared to those with lower arsenic levels. This finding reflects the widespread arsenic contamination of groundwater in Bangladesh and suggests that environmental pollutants may contribute to respiratory infection susceptibility through immunotoxic effects. However, as this finding is from a single study, replication in other arsenic-endemic areas would strengthen the evidence.

Other environment-related factors examined but not consistently associated with pneumonia across studies included the type of cooking fuel used, the presence of household pets, sanitation facilities, and exposure to traffic fumes. Brown et al. [36] specifically examined multiple environmental predictors of recurrent fast-breathing pneumonia in Pakistan and found that most environmental factors examined (including ventilation, fuel type, crowding, sanitation, and traffic exposure) were not significantly associated with recurrence, though poor housing quality was significant.

#### Socioeconomic risk factors.

Socioeconomic factors were examined in multiple studies, with housing quality and overall socioeconomic status emerging as significant risk factors. Ram et al. [37] found that children from households with tin roofs over living spaces were more likely to have pneumonia, with case children's living spaces being 54% more likely to have tin roofs compared to controls. Brown et al. [36] identified poor housing quality as a significant predictor of recurrent pneumonia episodes in Pakistan. Low socioeconomic status was associated with increased pneumonia risk in the Bangladesh study by Ram et al. [37].

Parental education, particularly maternal education, was examined in several studies with inconsistent findings. Gothankar et al. [35] reported significant associations between pneumonia and maternal awareness of pneumonia symptoms (OR = 0.36, 95% CI: 0.26-0.48) and of air pollution and overcrowding as risk factors (OR = 0.68, 95% CI: 0.52-0.88); however, the authors noted that the odds of pneumonia were higher among children of aware mothers, most likely because knowledge was gained after the child's illness, so these associations should not be interpreted as protective. However, formal maternal education level was not consistently associated with pneumonia across studies. Similarly, paternal occupation and father's education were examined in some studies but were not consistently identified as significant risk factors.

A summary of risk factor findings across all included studies, organized by variable category and number of studies identifying each factor, is presented in Table 2. The most consistently identified risk factors across multiple studies were young age (4 studies), malnutrition (3 studies), and incomplete immunization (2 studies). Synthesising across studies, three patterns emerge. First, the risk factors differ markedly in how modifiable they are. Young age was the most frequently reported association but is not itself amenable to intervention; it instead identifies the window (0–23 months) in which preventive effort must be concentrated. By contrast, malnutrition, incomplete immunisation, and indoor smoke exposure are directly actionable, and each was associated with a two- to four-fold increase in odds: underweight (AOR 2.3, 95% CI 1.4-3.9), partial immunisation (OR 2.41, 95% CI 1.06-5.49), indoor smoke-producing stoves (OR 3.76, 95% CI 1.20-11.82), and maternal smoking (AOR 2.5, 95% CI 1.1-5.7). Second, when ranked by the magnitude of association rather than by the number of studies reporting them, environmental exposures - particularly household air pollution - carried effect sizes at least as large as the nutritional and immunisation factors that currently dominate policy attention, suggesting that household air pollution is an under-prioritised target. Third, protective exposures were also actionable: adequate cross-ventilation of the living area was associated with lower odds of pneumonia (AOR 0.72, 95% CI 0.53-0.98), indicating that low-cost housing interventions may complement biomedical ones. Taken together, the evidence points to a modest number of high-yield, co-occurring targets - nutrition, immunisation coverage, and household air pollution - clustered in the first two years of life, rather than a long undifferentiated list of risks. It should be noted that these effect estimates derive from studies with heterogeneous designs, exposure definitions, and confounder adjustment, and are therefore not formally comparable; they are presented to indicate relative magnitude and consistency of direction, not as pooled estimates. Important evidence gaps also emerge: no included study quantified the interaction between undernutrition and household air pollution despite their frequent co-occurrence, no study from Sri Lanka, Afghanistan, or the Maldives met the inclusion criteria, and population-based aetiological data outside hospital settings remain scarce.

**Table 2:** Distribution of study Variables.

Variables	Significant risk factors	Number of studies identified these risk factors
1. Host related	1. Age of the children	4
	2. Partial/incomplete immunisation	2
	3. Malnutrition	3
	4. Health-related conditions/co-morbidity	2
	5. Gender of the child	1
	6. Home delivery	1
2. Environment related	1. Ventilation	1
	2. Overcrowding	1
	3. Smoking	1
	4. Smoke-producing fuel use	1
	5. Location of living areas	1
	6. Arsenic exposure	1
3 Socioeconomic statuses	1. Ethnicity/caste	1
	2. House	2
	3. Parents/maternal education /knowledge (reverse association)	1
	4. Overall poor socioeconomic status	1

### Aetiological agents of community-acquired pneumonia

Eight studies examined the aetiological agents of childhood CAP across five South Asian countries: Bangladesh (n = 2), Nepal (n = 2), India (n = 3), Pakistan (n = 1), and Bhutan (n = 1). These studies employed various diagnostic methods and specimen types to identify viral and bacterial pathogens. The findings are summarised in Table 3.

**Table 3:** Summary finding table of the studies reported aetiology.

	Author & year of publication	Study Design	Sample size	Specimen type	Lab Method	Findings for pneumonia cases				Findings for Control group
						Identified Pathogens	N (%)	Odds ratio	By age	N (%)
1	Hasan et al. (2006)	Cohort study	252	1. NPA (from 58 cases/93% of admitted cases)	ELISA	*Virus*	26/58(44.8)			
						*RSV*	21/58(36.2)			
						*PIV 2*	3/58(5.2)			
						*PIV3*	1/58(1.7)			
						*Influenza B*	1/58(1.7)			
				2. Blood N=48	Blood culture	*1. Bacteria isolation*	6/48(12.5)			
						*Pseudomonas aeruginosa*	1/48(2.1)			
						*Barnhamella catarrhalis*	1/48(2.1)			
						*S. aureus*	2/48(4.2)			
						*S. epidermidis*	1/48(2.1)			
						*S. pneumoniae*	1/48(2.1)			
						*No bacteria*	42/48(87.5)			
2	Brooks et al. (2021)	Case-control study	525 cases, 772 controls (219 CXR+ cases)	1. Blood	Blood culture	Bacteria:	1.5% positive			No bacteria detected
						*Salmonella typhi*	2(0.4%)			
						*E. coli (same child as K. pneumoniae)*	1(0.2%)			
						*K. pneumoniae*	1(0.2%)			
				2. Sputum	Sputum culture	*M.tuberculosis*	2(0.4%)			
				3. NP/OP Swab	PCR	*1. Virus:*	77.7% (estimated aetiologic fraction, CXR+ cases)			
						*RSV*	25.1%	12.9		3.2%
						*PIV 1*	4.6%	8.0		0.9%
						*hMPV*	8.7%	5.8		3.1%
						*Pneumococcus (S. pneumoniae, bacterium)*	67.6%	0.55		82.1%
						*Rhinovirus*	35.6%			25.4%
						*CMV*	29.7%			29.1%
						*Influenza*	1.9%			2.7%
3	Jullien et al. (2020)	Prospective cohort study	189	1. Blood	1. Blood culture (BacT/ALERT)	**Bacteria:** *S. pneumoniae*	2/148(1.4)			
						*Pseudomonas sp.*	2/148(1.4)			
						*E. coli*	1/148(0.7)			
						*Acinetobacter sp.*	1/148(0.7)			
						*S. typhi*	1/148(0.7)			
						*Serratia rubidaea*	1/148(0.7)			
					2. Blood RT-PCR	*S. pneumoniae*	1/148(0.7)			
				2. pleural fluid	Pleural fluid culture	*S. pneumoniae*	1/1(100)			
				3. Nasopharyngeal wash	RT-PCR	1. Bacteria: *Bordetella pertussis*	3/115(2.6)			
						*2.Virus:*				
						*At least 1 virus detected in NPW*	103/115(89.6)			
						*Single virus infection*	68/103(66)			
						*Mixed viral infection*	35/103(34)			
						*RSV*	52/115(45.2)			
						*Rhinovirus*	42/115(36.5)			
						*PIV virus*	19/115(16.5)			
						*Influenza virus*	16/115(13.9)			
						*Adenovirus*	8/115(7.0)			
						*Bocavirus*	6/115(5.2)			
						*hMPV*	4/115(3.5)			
						*Coronavirus*	2/115(1.7)			
4	Singh et al.,(2021)	Cross-sectional study	150	Blood NPS	Blood & NPS culture (blood, chocolate, MacConkey agar	*S. aureus*	22/150(14.7) Blood 28/150(18.7) NPS			
						*S. pneumoniae*	5/150(3.3) 9/150(6.0)			
						*E. coli*	8/150(5.3) 7/150(4.7)			
						*Klebsiella*	5/150(3.3) 7/150(4.7)			
						*CONS*	4/150(2.7) 5/150(3.3)			
						*Pseudomonas*	3(2.0) 4(2.7)			
5	Mathisen et al. (2010)	Case-control study	680 cases,680 controls	NPA	RT-PCR	Virus: *RSV*	66/680(9.7)			8/680(1.2%)
						*Influenza A*	20/680(2.9)			4/680(0.6%)
						*Influenza B*	20/680(2.9)			9/680(1.3%)
						*PIV TYPE 1*	28/680(4.1)			7/680(1.02%)
						*PIV type 2*	6/680(0.9)			3/680(0.5%)
						*PIV Type 3*	96/680(14.1)			12/680(1.8%)
						*hMPV*	23/680(3.4)			7/680(1.03%)
						Any virus	248/680(36.5)			48/680(7.1%)
6	Mathisen et al., (2009)	Cross-sectional study	2230	NPA	Multiplex RT-PCR	*RSV*	334/2219(15.1%)			
						*Influenzae A*	164/2219(7.4)			
						*PIV type 3*	129/2219(5.8)			
						*PIV type 1*	98/2219(4.4)			
						*hMPV*	93/2219(4.2)			
						*Influenzae B*	84/2219(3.8)			
						*PIV type 2*	17/2219(0.8)			
7	Bashir et al. (2017)	Observational study (active surveillance)	155	NP/OP swab	RT-PCR	Virus:	117/155 (75%)			
						> 1 virus detected	50/155 (32%)			
						*RSV -A*	69/155(44%)		2-6 months age group	
						*RSV-B*	35/155(23%)		2.1-6 months group	
						*Influenza-A*	38/155(24.5%)		6.1-12 months	
						*Influenza -B*	11/155(7%)			
						*Rhinovirus*	19/155(12%)			
						*Adenovirus*	13/155(8.4%)			
						*hMPV*	8/155(5.2%)			
8	Rajan et al. (2025)	Case-control study	34 cases, 32 controls	Tracheal aspirate, Nasopharyngeal swab, Non-bronchoscopic BAL	Multiplex PCR (TaqMan Array Card)	Virus: RSV; Bacteria: *S. pneumoniae* (the two most common pathogens)	RSV & *S. pneumoniae* most commonly detected; RSV significantly higher in cases; blood culture positive in 3/34 (8.8%: 2 S. aureus, 1 S. pneumoniae)	RSV (NP): 10.45 (1.25-88.25)		

#### Viral pathogens.

Viral pathogens were examined in seven of the eight aetiological studies, with respiratory syncytial virus (RSV) consistently emerging as the most frequently detected viral pathogen. RSV detection rates ranged from 9.7% to 45.2% across studies, with a mean detection rate of 29.2% (median 29.4%) across the seven studies reporting RSV detection. The highest RSV detection rate (45.2%) was reported in the Bhutan study by Jullien et al. [25], which used RT-PCR on nasopharyngeal wash specimens. The PERCH study site in Bangladesh reported RSV detection in 25.1% of chest radiograph-positive pneumonia cases [41]. Mathisen et al. [42] found RSV in 9.7% of cases in Nepal using RT-PCR on nasopharyngeal aspirates, though this study employed a case-control design and found RSV significantly more common in cases than controls. A larger cross-sectional study from the same Bhaktapur setting found RSV in 15.1% of 2,219 children with pneumonia [63]. A more recent case-control study from Vellore, South India, by Rajan et al. [62] similarly identified RSV as the most frequently detected pathogen in children aged 1–24 months intubated for community-acquired severe pneumonia, with RSV detected significantly more often in cases than controls in nasopharyngeal samples (odds ratio 10.45, 95% CI 1.25-88.25). This study additionally sampled the lower respiratory tract using tracheal aspirates and non-bronchoscopic bronchoalveolar lavage and used TaqMan Array Card multiplex PCR, providing specimens more representative of the aetiological agent.

RSV burden was notably higher in younger age groups. Bashir et al. [43] from Pakistan found that RSV-A was detected in 44% of pneumonia cases, with the highest detection rates in the 2–6 month age group. This age-specific pattern is consistent with global data showing that RSV-associated morbidity is highest in the first year of life, with incidence two to three times higher in infants compared to the overall under-five population [44]. The predominance of RSV across studies from different South Asian countries suggests that RSV represents a major target for preventive interventions in this region.

Rhinovirus was detected in three studies with variable detection rates: 36.5% in the Bhutan study [25], 35.6% in the Bangladesh PERCH study [41], and 12% in the Pakistan study [43]. The high rhinovirus detection rates, particularly in the Bhutan and Bangladesh studies, are notable and align with emerging evidence suggesting rhinovirus may be an underappreciated cause of lower respiratory tract infections in young children. However, interpretation of rhinovirus findings is complicated by its frequent detection in asymptomatic individuals, making attribution of causality challenging.

Parainfluenza viruses were detected in most studies examining viral pathogens. Detection rates ranged from 4.6% to 16.5%, with type 3 being the most commonly detected subtype. Mathisen et al. [42] found parainfluenza type 3 to be significantly associated with pneumonia (14.1% in cases versus 1.8% in controls). Influenza viruses A and B were detected in all viral studies, with combined detection rates ranging from 1.9% to 24.5%. The Pakistan study by Bashir et al. [43] reported the highest influenza detection rate, with influenza A found in 24.5% and influenza B in 7% of cases, highlighting influenza as an important vaccine-preventable cause of childhood pneumonia in this setting.

Human metapneumovirus (hMPV) was detected in four studies with rates ranging from 3.4% to 8.7%. The Bangladesh PERCH study found hMPV in 8.7% of cases with a significant association (odds ratio 5.8) compared to controls [41]. Adenovirus was detected in two studies at rates of 7.0-8.4%, though adenovirus was frequently found as a co-infecting pathogen rather than a sole pathogen. Coronavirus was detected in only one study at a low rate (1.7%), predating the SARS-CoV-2 pandemic. Bocavirus was detected in the Bhutan study at 5.2% [25]. Cytomegalovirus (CMV) was detected in the Bangladesh PERCH study at 29.7%, though the pathogenic significance of CMV detection in respiratory specimens from immunocompetent children remains unclear. Co-infections with multiple viral agents were common across studies. Jullien et al. [25] found that one-third of children with viral detection had mixed viral infections. This finding has implications for both diagnosis and treatment, as the contribution of individual pathogens to disease in the context of co-infection is difficult to determine.

#### Bacterial pathogens.

Bacterial pathogens were examined in five studies using blood culture, nasopharyngeal culture, sputum culture, and/or pleural fluid culture in four studies and molecular detection (multiplex PCR) in one study. Bacterial culture positivity was generally lower than viral detection, with blood culture positivity ranging from 1.5% to 31.3% across studies and below 13% in four of the five studies reporting it. This relatively low bacterial isolation rate may reflect several factors: prior antibiotic use before specimen collection, challenges in obtaining adequate blood volumes from young children, inherent limitations of culture-based diagnostics, and a genuine predominance of viral over bacterial aetiology in these populations [45,46].

The Bangladesh PERCH study reported the lowest blood culture positivity, at 1.5% [41]. After standardisation for age and severity, bacterial pathogens accounted for only 9.7% of the estimated aetiology in Bangladesh, compared with over 30% at all other PERCH sites except Kenya (16.8%); the authors suggested that earlier access to effective treatment in the study catchment areas may partly explain this. In contrast, the rural Bangladesh cohort study by Hasan et al. [47] found bacterial isolation in 12.5% of blood cultures. Singh et al. [48] reported the highest blood culture positivity, at 31.3% (47/150), with S. aureus the most common isolate.

The most commonly isolated bacterial pathogens across studies included *Streptococcus pneumoniae*, *Staphylococcus aureus, Escherichia coli, Klebsiella pneumoniae*, Pseudomonas species, and *coagulase-negative staphylococci*. The relative frequency of different bacterial pathogens varied across studies and specimen types. Singh et al. [48] from India found *S. aureus* to be the most commonly isolated organism in both blood cultures (14.7%) and *nasopharyngeal specimens* (18.7%), with particularly high proportions in younger children (3–12 months). S. pneumoniae was detected less frequently, possibly reflecting pneumococcal vaccine introduction in some study populations. In the case-control study by Rajan et al. [62] from South India, *S. pneumoniae* was, alongside RSV, one of the two most commonly detected pathogens, although its detection in nasopharyngeal samples did not differ significantly between cases and controls, in keeping with nasopharyngeal carriage at this age.

Jullien et al. [25] from Bhutan reported the most diverse range of bacterial pathogens, identifying *S. pneumoniae, Pseudomonas species, E. coli*, Acinetobacter species, Salmonella typhi, and Serratia rubidaea from blood cultures, and *Bordetella pertussis* from nasopharyngeal wash specimens. The detection of B. pertussis in respiratory samples, associated with high fatality rates, particularly in non-immunised young infants, highlights the continued importance of pertussis vaccination in this region. *Mycobacterium tuberculosis* was detected in induced sputum specimens in the Bangladesh PERCH study [41], representing an important cause of pneumonia that may be underdiagnosed in routine clinical practice.

## Discussion

This systematic review provides a comprehensive synthesis of evidence on risk factors and aetiological agents of community-acquired pneumonia in children under five years of age across South Asian countries. The review identified 16 high-quality studies from five countries, revealing a complex pattern of host, environmental, and socioeconomic risk factors, alongside a predominantly viral aetiological profile with RSV as the leading pathogen. These findings have important implications for pneumonia prevention strategies, clinical management, and research priorities in this high-burden region.

### Key findings and comparison with global evidence

The identification of malnutrition as a critical risk factor for childhood pneumonia aligns with extensive global evidence demonstrating the bidirectional relationship between undernutrition and infectious disease susceptibility [17,49]. Malnutrition impairs multiple components of immune function, including cell-mediated immunity, complement system activity, and mucosal barrier integrity, thereby increasing vulnerability to respiratory pathogens and reducing the capacity to mount effective immune responses [50]. A systematic review by Rice et al. [17] estimated that malnutrition is an underlying cause in over 50% of childhood infectious disease deaths in developing countries. Our finding that both underweight and wasting are associated with increased pneumonia risk in South Asian children underscores the importance of integrating nutritional interventions into pneumonia prevention strategies. South Asia has among the highest rates of childhood undernutrition globally, with stunting affecting approximately 38% of under-five children in the region [51], suggesting substantial potential for pneumonia reduction through nutritional improvements.

The association between incomplete immunisation and increased pneumonia risk reinforces the protective effect of vaccination programmes. Multiple randomised trials and systematic reviews have demonstrated significant reductions in pneumonia incidence following introduction of *Haemophilus influenzae* type b (Hib) and pneumococcal conjugate vaccines [52,53]. Theodoratou et al. [52] estimated that Hib vaccines could prevent 18% of radiologically-confirmed pneumonia cases, while pneumococcal conjugate vaccines could prevent 26% of such cases. Our finding that partially immunised children in South Asia have approximately 2.4-fold higher pneumonia odds compared to fully immunised children emphasises the importance of strengthening immunisation coverage and reducing vaccine hesitancy in the region. While Hib vaccine coverage has improved substantially across South Asia since its introduction in 2009, pneumococcal conjugate vaccine (PCV) introduction is more recent and incomplete across countries, suggesting potential for further pneumonia reduction as PCV coverage expands.

The higher pneumonia risk observed in younger children, particularly those under 24 months, is consistent with global patterns and reflects the immaturity of the developing immune system [54]. Young infants have limited immunological memory, reduced complement activity, and ongoing maturation of adaptive immune responses, making them particularly susceptible to respiratory pathogens [55]. This age-specific vulnerability has implications for targeting preventive interventions, including ensuring timely vaccination, promoting exclusive breastfeeding, and prioritising healthcare access for the youngest children.

Environmental risk factors, particularly household air pollution from biomass fuel combustion and tobacco smoke exposure, emerged as significant modifiable determinants of childhood pneumonia in this review. These findings align with a substantial body of evidence from LMICs demonstrating the respiratory health impacts of indoor air pollution [56,57]. The WHO estimates that household air pollution is responsible for approximately 44% of pneumonia deaths in children under five years in LMICs [58,59]. The mechanisms involve direct epithelial damage, impaired mucociliary clearance, oxidative stress, and immunomodulation, which increase susceptibility to respiratory infections [38]. Our finding that children in homes with indoor stoves have nearly fourfold higher odds of pneumonia highlights the potential impact of clean cooking interventions. Several South Asian countries have implemented programmes to promote liquefied petroleum gas (LPG) or improved cookstoves, though coverage and sustained use remain challenges.

The identification of arsenic exposure as a potential risk factor for pneumonia in Bangladesh represents a novel finding with important regional implications. Bangladesh has among the highest rates of groundwater arsenic contamination globally, with millions of people exposed to arsenic levels exceeding WHO guidelines [60]. Arsenic exposure has broad health and economic impacts in affected populations, including immunotoxic effects that may increase susceptibility to respiratory infections [60]. While this finding requires replication, it suggests that arsenic mitigation efforts in Bangladesh may yield respiratory health co-benefits beyond the well-recognised reductions in cancer and cardiovascular disease risk.

The predominance of viral pathogens, particularly RSV, in aetiological studies from South Asia is consistent with findings from the global PERCH study and other systematic reviews [15,26]. RSV is increasingly recognised as the most important viral cause of severe lower respiratory infections in young children worldwide, with an estimated 33 million episodes and 3.2 million hospitalisations annually [44]. Our finding that RSV was consistently the most frequently detected pathogen across South Asian studies, with detection rates as high as 45%, underscores the substantial burden of RSV-associated pneumonia in this region. The most recent evidence from South India reinforces this pattern, with RSV again emerging as the leading pathogen in severe, intubated cases [62]. Importantly, several RSV preventive interventions are now available or in advanced development, including maternal vaccines and long-acting monoclonal antibodies such as nirsevimab, both of which are now the subject of WHO immunisation recommendations for infants [61]. Accelerated implementation of these interventions in South Asian countries could substantially reduce the burden of childhood pneumonia.

The relatively low bacterial culture positivity rates observed in South Asian studies (1.5-31.3%, and below 13% in four of five studies) are notable and may reflect several factors. Prior antibiotic use before presentation, which is common in South Asia given the widespread availability of over-the-counter antibiotics, reduces the sensitivity of bacterial cultures [45]. Blood culture volumes achievable in young children are often suboptimal for bacterial detection. Additionally, the findings may reflect a genuine predominance of viral over bacterial aetiology in these populations, particularly in the context of increasing Hib and pneumococcal vaccine coverage. Nevertheless, bacterial pathogens, especially *S. pneumoniae* and *S. aureus*, remain important causes of severe pneumonia requiring appropriate antimicrobial therapy, and treatment guidelines must account for regional patterns of bacterial aetiology.

### Strengths and limitations

This systematic review has several strengths. To our knowledge, it represents the first comprehensive systematic review examining both risk factors and aetiological agents of CAP specifically in South Asian children under five years. The rigorous methodology, including systematic searches across four databases, the application of explicit inclusion and exclusion criteria, quality assessment using a validated tool, and the exclusion of poor-quality studies, enhances the reliability of the findings. The inclusion of studies from five South Asian countries provides a broad regional perspective, while the categorisation of risk factors into host, environmental, and socioeconomic domains facilitates translation to prevention strategies.

Several limitations must be acknowledged. First, the search was restricted to English-language publications, which may have excluded relevant studies published in regional languages (for example, Hindi, Bengali, Urdu, or Nepali) and could introduce language bias; however, the great majority of peer-reviewed research from the region is published in English, and no non-English record identified during screening met the other eligibility criteria. Second, substantial heterogeneity in study designs, pneumonia definitions, risk factor measurements, and diagnostic methods precluded meta-analysis and limits direct comparison across studies. The variation in pneumonia case definitions—ranging from clinical WHO criteria alone to radiologically-confirmed pneumonia—introduces diagnostic heterogeneity that may affect both risk factor associations and aetiological findings. Third, most aetiological studies were hospital-based, potentially overrepresenting severe cases and underrepresenting the full spectrum of community-acquired disease. Hospital-based studies may also miss pathogens causing milder illnesses that do not prompt healthcare-seeking. Fourth, the included studies span a 24-year period (2000–2024), during which conjugate vaccination programmes against Haemophilus influenzae type b and Streptococcus pneumoniae were progressively introduced across South Asian countries [13], alongside evolving healthcare systems and socioeconomic conditions. The majority of the aetiological studies included in this review were conducted before, or during the early phase of, national pneumococcal conjugate vaccine (PCV) introduction in their respective countries; consequently, the pooled aetiological profile reported here should be interpreted as a time-averaged snapshot rather than the current picture, and may overstate the contemporary burden of vaccine-preventable bacterial pathogens relative to settings with mature PCV programmes. Fifth, although Sri Lanka, Afghanistan, and the Maldives were eligible under our inclusion criteria, no studies from these countries met the full eligibility criteria and none were included in the final synthesis. This is a notable gap, particularly for Sri Lanka, which has achieved comparatively low under-five pneumonia mortality relative to other countries in the region, an outcome attributed in the wider literature to its universal, decentralised primary healthcare system. The absence of Sri Lankan data means this review cannot examine how cross-country variation in health-system organisation and access may modify the risk factors and aetiological patterns identified here; we recommend that future primary research and reviews specifically target Sri Lanka, Afghanistan, and the Maldives, both to fill this evidence gap and to enable comparison between low- and higher-mortality health systems within South Asia. Sixth, although a random 10% sample of full-text screening decisions was independently cross-checked by a second, blinded reviewer, with disagreements resolved by consensus, the review was otherwise conducted by a single primary reviewer for an academic dissertation. Full independent dual screening of all records was not feasible within the scope of this dissertation-based review, and the extent and direction of any residual selection bias arising from the remaining unchecked decisions cannot be determined; we recommend that future updates of this review adopt full dual independent screening to minimise this risk. Seventh, the two studies by Mathisen et al. [42,63] were conducted in the same population in Bhaktapur, Nepal, with overlapping enrolment periods, so some children may contribute to both; their findings were therefore interpreted together rather than as independent evidence. Finally, publication bias may have affected findings, as studies with statistically significant results may be more likely to be published than those with null findings.

### Implications for policy and practice

The findings of this review support multi-pronged, integrated approaches to childhood pneumonia prevention in South Asia. First, addressing malnutrition through nutrition-specific interventions (breastfeeding promotion, complementary feeding support, micronutrient supplementation) and nutrition-sensitive approaches (improving food security, women's empowerment, water and sanitation) should be priorities given the strong and consistent association with pneumonia risk. Second, strengthening immunization programmes to achieve high coverage with existing vaccines (Hib, PCV, pertussis, influenza) and introducing new vaccines (RSV) as they become available could substantially reduce pneumonia incidence. Third, interventions to reduce household air pollution through clean cooking technologies and smoking cessation support address modifiable environmental risks. Fourth, broader socioeconomic development, improved housing conditions, and parental education contribute to creating environments protective against childhood pneumonia.

For clinical practice, the predominance of viral aetiology suggests that most childhood CAP cases do not require antibiotic treatment, supporting efforts to reduce unnecessary antibiotic use. However, given the difficulty of distinguishing viral from bacterial pneumonia clinically and the serious consequences of untreated bacterial infection, clinical guidelines must balance antimicrobial stewardship with appropriate treatment of bacterial cases. Development of affordable, accurate point-of-care diagnostics could help differentiate viral from bacterial pneumonia and guide treatment decisions. The aetiological findings translate into three concrete regional priorities. For targeted prevention, the consistent predominance of RSV - the leading pathogen in seven of the eight aetiological studies, with a mean detection rate of 29.2% and a significant case-control association in the most recent evidence from South India - makes RSV-directed prophylaxis the single highest-yield addition to the current preventive portfolio. Maternal RSV vaccination and long-acting monoclonal antibodies (for example, nirsevimab) protect precisely the 0–23-month window that this review identifies as the period of peak risk, and their cost-effectiveness in South Asian settings warrants urgent evaluation. In parallel, the persistence of *Streptococcus pneumoniae* as the leading bacterial pathogen, in a region where PCV coverage remains incomplete (only 28% of cases in the most recent study had received PCV), argues for accelerating PCV introduction and coverage, while monitoring serotype replacement. For diagnostic priorities, the low yield of blood culture (positive in under 9% of severe cases) confirms that culture-based diagnosis alone substantially underestimates bacterial contribution; investment in affordable multiplex molecular platforms, and in specimen types that better represent the lower respiratory tract, would materially improve aetiological attribution. Critically, the finding that potential pathogens are also detected in the upper airway of children without pneumonia means that molecular detection must be interpreted against control data, and that nasopharyngeal positivity alone should not be equated with causation. For surveillance, the region currently lacks standardised, population-based pneumonia aetiology surveillance; establishing sentinel sites that apply uniform WHO case definitions, collect paired upper and lower respiratory specimens, and report RSV and pneumococcal detection alongside vaccination status would allow the impact of PCV and forthcoming RSV interventions to be tracked, and would close the surveillance gap that this review identifies.

### Recommendations for future research

This review identifies several priorities for future research. First, large-scale, community-based aetiological studies employing standardised diagnostic methods across diverse South Asian settings are needed to better characterise the pathogen landscape beyond hospital-based populations. Second, implementation research evaluating integrated interventions that address multiple risk factors simultaneously is needed to inform programmatic scale-up. Third, standardised approaches to measuring household ventilation, crowding, and air pollution exposures would facilitate comparison across studies. Fourth, research on the impact of RSV preventive interventions as they are introduced in South Asian countries will be important for evaluating their effectiveness in real-world settings. Fifth, investigation of emerging environmental exposures, including arsenic and outdoor air pollution, and their interactions with traditional risk factors warrants attention. Sixth, economic evaluations of pneumonia prevention interventions in South Asian contexts would inform resource allocation decisions.

## Conclusions

This systematic review demonstrates that childhood community-acquired pneumonia in South Asia results from a complex interplay of modifiable host, environmental, and socioeconomic risk factors. Malnutrition, incomplete immunisation, and young age represent the most critical and consistently identified risk factors amenable to public health intervention. Indoor smoke exposure from biomass fuels and tobacco, poor household ventilation, and socioeconomic disadvantage contribute to additional risk. The predominance of viral pathogens, particularly respiratory syncytial virus, accounting for over one-quarter of cases, underscores the urgent need for accelerated development and deployment of RSV vaccines and preventive monoclonal antibodies in South Asian countries. Reducing the substantial childhood pneumonia burden in South Asia will require integrated approaches that address nutrition, immunisation, the household environment, and socioeconomic determinants. Policymakers should prioritise evidence-based interventions targeting these modifiable risk factors while strengthening health systems for pneumonia case management. Continued surveillance and research are essential to monitor the evolving epidemiology and evaluate the effectiveness of interventions in this high-burden region.

## Supporting information

S1 Text Search terms including MeSH terms. Search strategy used for Web of Science, PubMed, MEDLINE and Global Health (original search June–July 2024; updated search January 2026).
(DOCX)

S1 Table CCAT quality appraisal of the included studies. Category and total scores for all appraised studies.
(DOCX)

S2 Table Characteristics of included studies: risk factors. (DOCX)

S3 Table Characteristics of included studies: aetiology. (DOCX)

S1 Data Study-level extracted data underlying all results, tables and figures (risk-factor effect estimates, pathogen detection counts and proportions, CCAT scores and PRISMA counts). (XLSX)

S1 File PRISMA 2020 checklist. From: Page MJ, McKenzie JE, Bossuyt PM, Boutron I, Hoffmann TC, Mulrow CD, et al. The PRISMA 2020 statement: an updated guideline for reporting systematic reviews. BMJ 2021;372:n71. https://doi.org/10.1136/bmj.n71. This work is licensed under CC BY 4.0. To view a copy of this license, visit https://creativecommons.org/licenses/by/4.0/.
(DOCX)
