# Supplementary material for: Risk factors and aetiological agents of community-acquired pneumonia in children under five years in South Asia: A systematic review
Source: PLOS Glob Public Health. 2026 Nov 10;6(11):e0006388. doi: 10.1371/journal.pgph.0006388 (PMC13750549; doi:10.1371/journal.pgph.0006388)
Supplement: Supplementary file 1 [file pgph.0006388.s001.docx]

***S1 Text. Search terms including MeSH terms.***

***Search terms***

| Aetiology* OR etiology* OR “causative organism*” OR “risk factor*” OR predictor* | AND | “Community acquired pneumonia*” OR pneumonia* OR “lower respiratory tract infection*” | AND | “Children under five*” OR children*OR Infant* OR paediatric*OR “young infant*” OR neonate* | AND | South Asia* |
| --- | --- | --- | --- | --- | --- | --- |

***MeSH terms***

| “Risk factor*” | Pneumonia* | Paediatric*  OR child* OR infant* | “Asia Southern” |
| --- | --- | --- | --- |

*Note: The original search was performed between June and July 2024 (studies published January 2000 - July 2024). An updated search using the same terms, MeSH headings and databases was conducted in 2026 to identify eligible studies published after July 2024 (studies published August 2024 to January 2026).*
