# Supplementary material for: Risk factors and aetiological agents of community-acquired pneumonia in children under five years in South Asia: A systematic review
Source: PLOS Glob Public Health. 2026 Nov 10;6(11):e0006388. doi: 10.1371/journal.pgph.0006388 (PMC13750549; doi:10.1371/journal.pgph.0006388)
Supplement: Supplementary file 2 [file pgph.0006388.s002.docx]

***S1 Table. CCAT quality appraisal of all 22 appraised studies (studies scoring below 75% were excluded from the synthesis)***

|  | Case-control | | | | | | | | | Cohort | | | | Cross-sectional | | | | | Secondary analysis survey | Case series | Active surveillance study | Hospital-based observational study |
| --- | --- | --- | --- | --- | --- | --- | --- | --- | --- | --- | --- | --- | --- | --- | --- | --- | --- | --- | --- | --- | --- | --- |
|  | Karki | Mahalanabis | Ram | Fatmi | Brown | George | Brook | Mathisen | Rajan | Goyal | Mostafa | Hasan | Jullien | Gothankar | Dhimal | Mathisen | Yadav | Singh | Dharel | Divyarani | Bashir | Agarwal |
| Preliminaries | 4 | 4 | 3 | 4 | 4 | 5 | 4 | 5 | 4 | 4 | 3 | 4 | 5 | 5 | 4 | 5 | 3 | 4 | 4 | 3 | 4 | 3 |
| Introduction | 4 | 4 | 4 | 4 | 4 | 4 | 5 | 4 | 4 | 4 | 4 | 4 | 5 | 5 | 4 | 5 | 5 | 5 | 5 | 3 | 4 | 4 |
| Design | 4 | 4 | 4 | 4 | 4 | 4 | 4 | 4 | 4 | 4 | 3 | 3 | 5 | 4 | 3 | 4 | 3 | 4 | 4 | 2 | 4 | 3 |
| Sampling | 3 | 3 | 4 | 4 | 4 | 4 | 5 | 4 | 3 | 5 | 2 | 4 | 4 | 4 | 3 | 4 | 3 | 3 | 5 | 2 | 4 | 4 |
| Data collection | 5 | 3 | 4 | 4 | 4 | 5 | 5 | 4 | 5 | 5 | 2 | 4 | 5 | 5 | 4 | 4 | 4 | 4 | 4 | 2 | 4 | 3 |
| Ethical matter | 4 | 0 | 2 | 1 | 4 | 5 | 4 | 4 | 4 | 4 | 1 | 4 | 5 | 5 | 2 | 4 | 3 | 4 | 4 | 1 | 4 | 2 |
| Result | 4 | 4 | 4 | 4 | 4 | 4 | 5 | 5 | 4 | 5 | 4 | 4 | 5 | 4 | 3 | 4 | 4 | 4 | 5 | 2 | 5 | 4 |
| Discussion | 3 | 3 | 5 | 5 | 4 | 4 | 5 | 4 | 4 | 4 | 2 | 4 | 5 | 5 | 3 | 4 | 4 | 3 | 5 | 2 | 4 | 3 |
| Total (40) | 31 | 25 | 30 | 30 | 32 | 35 | 37 | 34 | 32 | 35 | 21 | 31 | 39 | 37 | 26 | 34 | 29 | 31 | 36 | 17 | 33 | 26 |
| % | 78 | 63 | 75 | 75 | 80 | 88 | 93 | 85 | 80 | 88 | 53 | 78 | 98 | 93 | 65 | 85 | 73 | 78 | 90 | 43 | 83 | 65 |
