# Supplementary material for: Risk factors and aetiological agents of community-acquired pneumonia in children under five years in South Asia: A systematic review
Source: PLOS Glob Public Health. 2026 Nov 10;6(11):e0006388. doi: 10.1371/journal.pgph.0006388 (PMC13750549; doi:10.1371/journal.pgph.0006388)
Supplement: Supplementary file 3 [file pgph.0006388.s003.docx]

***S2 Table. Characteristics of studies appraised for risk factors (studies marked as excluded scored below 75% on CCAT appraisal)***

| **Author & year** | **Study type** | **Age of participant** | **Journal**  **& Country of the study** | **Definition of Pneumonia used in studies** | **Potential Risk factors the study looked for** |
| --- | --- | --- | --- | --- | --- |
| 1. Mostafa et al., 2022 (excluded after CCAT appraisal) | Prospective clinical study | 1month-5 years | AIMDR.  Bangladesh | WHO ARI control program defined clinical criteria for pneumonia | 1. Kucha house,  2. Poor sanitation  3. Crowding  4. Cooking with fuel other than LPG  6. Malnutrition  7. Female child  8. High respiratory rate  9. Anaemia  10. Low socioeconomic status |
| 2.Ram et al.,2014 | Case-control study | Under five years | The American Journal of Tropical Medicine and Hygiene.  Bangladesh. | Clinical and radiological criteria for pneumonia | 1. Ventilation  2. Overcrowding  3. Male sex  4. Low socioeconomic status  5. Tin roof above living space  6. High level of particulate matter in indoor air. |
| 3. George et al.,2015 | Case-control study | 28days-59months. | Environmental Health.  Bangladesh | WHO criteria for severe and very severe pneumonia | Arsenic exposure (both water and dietary exposure) from elevated arsenic levels in drinking water. |
| 4.Gothankar et al.2018 | Cross-sectional | Under five years | BMC Public Health.  India | WHO defined clinical criteria. | 1. ventilation status  2. overcrowding  3. type of fuel used for cooking  4. indoor smoking by family members  5. Immunisation status  6. Nutritional status of children  7. knowledge, attitude, and practices of the mother with pneumonia.  8. Breastfeeding |
| 5. Mahalanabis et al., 2002 (excluded after CCAT appraisal) | Case-control study | 2-35 months | Epidemiology and Infection.  India | WHO ARI algorithm for pneumonia | 1. Families with large numbers of children  2. Solid fuel use  3. Infant delivered at home  4. History of asthma  5.Poor economic status  6. Keeping large animal  7. Lack of parents education |
| 6. Goyal et al., 2021 | Prospective cohort study | 2-59 months | Indian Paediatrics.  India | WHO criteria | 1. Overcrowding  2. Solid fuel used in household  3. Incomplete immunisation  4. Undernutrition  5. Male gender  6. Younger child.  7. lack of Exclusive Breast Feeding  8. low maternal education |
| 7.Divyarani et al., 2014 (excluded after CCAT appraisal) | Observational study (case series) | Under 5 years | International journal of current microbiology and applied science.  India | WHO criteria | 1. Overcrowding due to joint family  2. Delivery by caesarean section,  3. low birth weight  4. Failure to initiate breastfeeding within  4 hours of birth  5. Income, education, and occupation of  Parents |
| 8. Karki et al., 2014 | Case-control study | 1month-5years | Kathmandu University Medical Journal.  Nepal | Clinical criteria for pneumonia | 1. Poor ventilation  2. Presence of smoky chulo/stove  3. Parent smoking  4. Partial immunisation  5. Maternal age  6. Age of child, birthweight, a child  attending a childcare centre  7. Breastfeeding  8. co-morbidities like diarrhoea |
| 9. Dharel et al., 2022 | Secondary analysis of the survey | 0-59 months | BMC Public Health.  Nepal | CB-IMCI criteria for pneumonia | 1. Current smoking mothers  2. Stunted, wasted, and underweight children.  3. Younger age (0-23 months)  4. Children from the rural area |
| 10. Dhimal et al.,2010 (excluded after CCAT appraisal) | Cross-sectional study | Under 59 months | Journal of Nepal Health Research Council.  Nepal | CB-IMCI diagnosis protocol | Indoor smoke produced by solid biomass fuel |
| 11. Fatmi and white,2002 | Case-control study | Under 5 years | International journal of infectious disease.  Pakistan | WHO ARI guideline | 1. Heating oil/wood  2. Passive smoking  3. lack of immunisation  4. malnutrition(wasting)  5. Younger age.  6. Children born at home.  7. Colostrum  8. Previous history of pneumonia and chest infections in the  neonatal period  9. The education of the parent and  occupation of the father. |
| 12. Brown et al.2020 | Case-control study | 2-60 months | BMJ.  Pakistan | WHO defined fast breathing pneumonia without chest indrawing | 1. Inadequate ventilation  2. Poor housing quality. |
