# Supplementary material for: Risk factors and aetiological agents of community-acquired pneumonia in children under five years in South Asia: A systematic review
Source: PLOS Glob Public Health. 2026 Nov 10;6(11):e0006388. doi: 10.1371/journal.pgph.0006388 (PMC13750549; doi:10.1371/journal.pgph.0006388)
Supplement: Supplementary file 4 [file pgph.0006388.s004.docx]

***S3 Table. Characteristics of studies appraised for aetiology (studies marked as excluded scored below 75% on CCAT appraisal)***

| **Author &year** | **Study type** | **Age of participants** | **Journal & Country of the study** | **Definition of Pneumonia used in studies** | **Specimen type** | **Aetiological agents identified** |
| --- | --- | --- | --- | --- | --- | --- |
| 1. Hasan et al.,2006 | Cohort study | 0-24  months | Scandinavian Journal of Infectious Diseases.  Bangladesh | WHO criteria | 1. NPA (from fifty-eight cases)  2. Blood (from  48 cases) | **Virus**  *RSV, Parainfluenza2, Parainfluenza3, Influenza*  **Bacteria**  *Pseudomonas aeruginosa,*  *Barnhamella catarrhalis,*  *S. aureus, S. epidermidis*  *S. pneumoniae* |
| 2. Brooks et al.,2021 | Case-control study | 1-59  months | The Paediatric Infectious Disease Journal.  Bangladesh | Modified WHO criteria 2005 | 1. Blood  2. Sputum  3. NP/OP swab | **Bacteria:**  *Salmonella typhi, E. coli, K. pneumoniae,* M. tuberculosis, S. pneumoniae (NP/OP PCR)  **Virus:**  *RSV, Parainfluenza Human, metapneumovirus, Rhinovirus, CMV, Influenza* |
| 3.Jullien et al.,2020 | prospective cohort study | 2-59  months | International  journal of infectious disease.  Bhutan | WHO defined pneumonia | 1. Blood  2. pleural fluid  3. Nasopharyngeal wash | **Bacteria:**  *S. pneumoniae, Pseudomonas sp., E. coli*, *Acinetobacter sp., S. typhi*, *Serratia rubidaea*, *Bordetella pertussis*  **Virus:**  *RSV, Rhinovirus, Parainfluenza virus,*  *Influenza virus, Adenovirus, Bocavirus,*  *Human Metapneumovirus, Coronavirus* |
| 4.  Agarwal et al.,2009 (excluded after CCAT appraisal) | hospital-based observational study | 1-59 months | Tropical Doctor.  India | WHO criteria | Blood and NPA | *M.pneumoniae* |
| 5.  Yadav et al.,2021 (excluded after CCAT appraisal) | prospective cross-sectional study | 2-60 months | Tropical doctors.  India | Revised WHO Criteria 2014 | Blood and Nasopharyngeal swab | *Staphylococcus aureus, Streptococcus pneumoniae, E. coli, K. pneumoniae, Pseudomonas, H. influenzae, CONS,* *Citrobacter koseri, Chlamydia pneumoniae* |
| 6. Singh et al.,2021 | A cross-sectional study | 3-59 months | Asian Journal of Medical Sciences.  India | WHO criteria | NPS and blood | *S aureus, S. pneumoniae, E. coli, Klebsiella, Coagulase negative Staphylococci (CONS) and Pseudomonas* |
| 7.  Mathisen et al.,2010 | Case-control study | 2-35  Months | The paediatric Infectious disease journal.  Nepal | WHO ARI algorithm for pneumonia | Nasopharyngeal aspirate | **Virus:**  *RSV, Influenza A, Influenza B, PIV TYPE 1, PIV Type 2, PIV Type 3, hMPV* |
| 8.  Mathisen et al.,2009 | cross-sectional study | 2-35 months | BMC Medicine.  Nepal | WHO criteria | NPA | *RSV, Influenza A virus, PIV3, PIV 1, hMPV, Influenza B, PIV type 2* |
| 9. Bashir et al.,2017 | Observational study (active surveillance) | <2  years | Archives of Virology.  Pakistan | WHO criteria | Nasopharyngeal or oropharyngeal swab | **Virus:**  *RSV -A, RSV-B, Influenza-A, Influenza -B, Rhinovirus, Adenovirus, hMPV* |
| 10. Rajan et al.,2025 | Case-control study | 1-24 months | American Journal of Tropical Medicine and Hygiene. India | Community-acquired severe pneumonia requiring intubation (WHO-based clinical criteria) | Tracheal aspirate, Nasopharyngeal swab, Non-bronchoscopic BAL | Virus: RSV Bacteria: S. pneumoniae (two most commonly detected pathogens) |
