# Supplementary material for: Risk factors and aetiological agents of community-acquired pneumonia in children under five years in South Asia: A systematic review
Source: PLOS Glob Public Health. 2026 Nov 10;6(11):e0006388. doi: 10.1371/journal.pgph.0006388 (PMC13750549; doi:10.1371/journal.pgph.0006388)
Supplement: Supplementary file 5 [file pgph.0006388.s006.docx]

**S1 PRISMA Checklist. PRISMA 2020 checklist**

**Title:** Risk Factors and Aetiological Agents of Community-Acquired Pneumonia in Children Under Five Years in South Asia: A Systematic Review

**Authors:** Sumaya Ferdowsi, Md Mamunur Rashid Sheikh

| **Section and Topic** | **Item #** | **Checklist item** | **Location where item is reported** |
| --- | --- | --- | --- |
| **TITLE** |  |  |  |
| Title | 1 | Identify the report as a systematic review. | Title page (title) |
| **ABSTRACT** |  |  |  |
| Abstract | 2 | See the PRISMA 2020 for Abstracts checklist. | Abstract |
| **INTRODUCTION** |  |  |  |
| Rationale | 3 | Describe the rationale for the review in the context of existing knowledge. | Introduction |
| Objectives | 4 | Provide an explicit statement of the objective(s) or question(s) the review addresses. | Introduction (final paragraph) |
| **METHODS** |  |  |  |
| Eligibility criteria | 5 | Specify the inclusion and exclusion criteria for the review and how studies were grouped for the syntheses. | Methods – Eligibility Criteria |
| Information sources | 6 | Specify all databases, registers, websites, organisations, reference lists and other sources searched or consulted to identify studies. Specify the date when each source was last searched or consulted. | Methods – Information Sources and Search Strategy |
| Search strategy | 7 | Present the full search strategies for all databases, registers and websites, including any filters and limits used. | Methods – Information Sources and Search Strategy; S1 Text |
| Selection process | 8 | Specify the methods used to decide whether a study met the inclusion criteria of the review, including how many reviewers screened each record and each report retrieved, whether they worked independently, and if applicable, details of automation tools used in the process. | Methods – Study Selection Process |
| Data collection process | 9 | Specify the methods used to collect data from reports, including how many reviewers collected data from each report, whether they worked independently, any processes for obtaining or confirming data from study investigators, and if applicable, details of automation tools used in the process. | Methods – Data Extraction |
| Data items | 10a | List and define all outcomes for which data were sought. Specify whether all results that were compatible with each outcome domain in each study were sought (e.g. for all measures, time points, analyses), and if not, the methods used to decide which results to collect. | Methods – Data Extraction |
|  | 10b | List and define all other variables for which data were sought (e.g. participant and intervention characteristics, funding sources). Describe any assumptions made about any missing or unclear information. | Methods – Data Extraction |
| Study risk of bias assessment | 11 | Specify the methods used to assess risk of bias in the included studies, including details of the tool(s) used, how many reviewers assessed each study and whether they worked independently, and if applicable, details of automation tools used in the process. | Methods – Quality Assessment; S1 Table |
| Effect measures | 12 | Specify for each outcome the effect measure(s) (e.g. risk ratio, mean difference) used in the synthesis or presentation of results. | Methods – Data Extraction (odds ratios, 95% CIs, p-values; pathogen detection rates) |
| Synthesis methods | 13a | Describe the processes used to decide which studies were eligible for each synthesis (e.g. tabulating the study intervention characteristics and comparing against the planned groups for each synthesis (item #5)). | Methods – Data Synthesis and Analysis |
|  | 13b | Describe any methods required to prepare the data for presentation or synthesis, such as handling of missing summary statistics, or data conversions. | Methods – Data Synthesis and Analysis |
|  | 13c | Describe any methods used to tabulate or visually display results of individual studies and syntheses. | Methods – Data Synthesis and Analysis; Tables 1–3 |
|  | 13d | Describe any methods used to synthesize results and provide a rationale for the choice(s). If meta-analysis was performed, describe the model(s), method(s) to identify the presence and extent of statistical heterogeneity, and software package(s) used. | Methods – Data Synthesis and Analysis (narrative synthesis; meta-analysis not appropriate) |
|  | 13e | Describe any methods used to explore possible causes of heterogeneity among study results (e.g. subgroup analysis, meta-regression). | Methods – Data Synthesis and Analysis |
|  | 13f | Describe any sensitivity analyses conducted to assess robustness of the synthesized results. | N/A (no sensitivity analyses conducted) |
| Reporting bias assessment | 14 | Describe any methods used to assess risk of bias due to missing results in a synthesis (arising from reporting biases). | Not formally assessed; acknowledged in Discussion – Strengths and Limitations |
| Certainty assessment | 15 | Describe any methods used to assess certainty (or confidence) in the body of evidence for an outcome. | N/A (certainty of evidence not formally assessed) |
| **RESULTS** |  |  |  |
| Study selection | 16a | Describe the results of the search and selection process, from the number of records identified in the search to the number of studies included in the review, ideally using a flow diagram. | Results – Study Selection; Fig 1 |
|  | 16b | Cite studies that might appear to meet the inclusion criteria, but which were excluded, and explain why they were excluded. | Results – Study Selection; Fig 1 |
| Study characteristics | 17 | Cite each included study and present its characteristics. | Results – Study Characteristics; S2 Table; S3 Table |
| Risk of bias in studies | 18 | Present assessments of risk of bias for each included study. | Results – Quality Assessment Results; S1 Table |
| Results of individual studies | 19 | For all outcomes, present, for each study: (a) summary statistics for each group (where appropriate) and (b) an effect estimate and its precision (e.g. confidence/credible interval), ideally using structured tables or plots. | Results – Risk Factors for Community-Acquired Pneumonia (Table 1); Aetiological Agents of Community-Acquired Pneumonia (Table 3) |
| Results of syntheses | 20a | For each synthesis, briefly summarise the characteristics and risk of bias among contributing studies. | Results – Risk Factors for Community-Acquired Pneumonia; Aetiological Agents of Community-Acquired Pneumonia |
|  | 20b | Present results of all statistical syntheses conducted. If meta-analysis was done, present for each the summary estimate and its precision (e.g. confidence/credible interval) and measures of statistical heterogeneity. If comparing groups, describe the direction of the effect. | Results – Risk Factors for Community-Acquired Pneumonia (Table 2); Viral Pathogens; Bacterial Pathogens |
|  | 20c | Present results of all investigations of possible causes of heterogeneity among study results. | Discussion – Key Findings and Comparison with Global Evidence; Strengths and Limitations |
|  | 20d | Present results of all sensitivity analyses conducted to assess the robustness of the synthesized results. | N/A (no sensitivity analyses conducted) |
| Reporting biases | 21 | Present assessments of risk of bias due to missing results (arising from reporting biases) for each synthesis assessed. | Not formally assessed; acknowledged in Discussion – Strengths and Limitations |
| Certainty of evidence | 22 | Present assessments of certainty (or confidence) in the body of evidence for each outcome assessed. | N/A (certainty of evidence not formally assessed) |
| **DISCUSSION** |  |  |  |
| Discussion | 23a | Provide a general interpretation of the results in the context of other evidence. | Discussion – Key Findings and Comparison with Global Evidence |
|  | 23b | Discuss any limitations of the evidence included in the review. | Discussion – Strengths and Limitations |
|  | 23c | Discuss any limitations of the review processes used. | Discussion – Strengths and Limitations |
|  | 23d | Discuss implications of the results for practice, policy, and future research. | Discussion – Implications for Policy and Practice; Recommendations for Future Research |
| **OTHER INFORMATION** |  |  |  |
| Registration and protocol | 24a | Provide registration information for the review, including register name and registration number, or state that the review was not registered. | Methods – Protocol and Registration (review not registered) |
|  | 24b | Indicate where the review protocol can be accessed, or state that a protocol was not prepared. | Methods – Protocol and Registration |
|  | 24c | Describe and explain any amendments to information provided at registration or in the protocol. | N/A (no registered protocol) |
| Support | 25 | Describe sources of financial or non-financial support for the review, and the role of the funders or sponsors in the review. | Declarations – Financial Disclosure |
| Competing interests | 26 | Declare any competing interests of review authors. | Declarations – Competing Interests |
| Availability of data, code and other materials | 27 | Report which of the following are publicly available and where they can be found: template data collection forms; data extracted from included studies; data used for all analyses; analytic code; any other materials used in the review. | Declarations – Availability of data and materials; Supporting Information (S1 Text, S1–S3 Table) |

*From: Page MJ, McKenzie JE, Bossuyt PM, Boutron I, Hoffmann TC, Mulrow CD, et al. The PRISMA 2020 statement: an updated guideline for reporting systematic reviews. BMJ 2021;372:n71. doi: 10.1136/bmj.n71*

*This work is licensed under CC BY 4.0. To view a copy of this license, visit https://creativecommons.org/licenses/by/4.0/*
