# Supplementary material for: Risk factors and aetiological agents of community-acquired pneumonia in children under five years in South Asia: A systematic review
Source: PLOS Glob Public Health. 2026 Nov 10;6(11):e0006388. doi: 10.1371/journal.pgph.0006388 (PMC13750549; doi:10.1371/journal.pgph.0006388)
Supplement: Supplementary file 6 [file pgph.0006388.s008.docx]

**Response to the Editor and Reviewer Comments**

**Manuscript ID: PGPH-D-26-01392**

Dear Dr Saravanos,

Thank you for the opportunity to revise our manuscript. We are grateful to you and the reviewer for the constructive comments, which have helped us improve the manuscript. Our point-by-point responses are provided in the table below. All corresponding changes are highlighted in the “Revised Manuscript with Track Changes” file.

| **No.** | **Comment** | **Author’s Response** |
| --- | --- | --- |
| **A. Journal Requirements** | | |
| **A1** | We note that the most recent literature search was completed over a year before the manuscript was submitted to the journal. Systematic and scoping reviews should provide an up-to-date overview of the research topic covered. Therefore, we request that you please update your searches as part of your revision to ensure that the most up-to-date literature is included in your review, or provide scientific justification for not doing so. Please ensure you mention this in your response to reviewers, and discuss whether this has any impact on the Results and Conclusions of the study. | We thank the Editor for this important observation and fully agree that a systematic review should reflect the most current evidence. We have therefore updated our search rather than seeking a justification for not doing so.  Using the identical search strategy, MeSH terms, and eligibility criteria as the original search, we re-ran the search across all four databases (MEDLINE, PubMed, Global Health, and Web of Science) to cover the period **August 2024 to January 2026**. This identified **204 additional records** (Web of Science = 13, PubMed = 75, MEDLINE = 54, Global Health = 62). After removing 147 duplicates, 57 records were screened, of which 49 were excluded at title and abstract. Of the 8 full-text articles assessed, 7 were excluded (3 without separate under-five data; 2 without pneumonia-specific data; 2 reporting different outcomes).  **One study met all inclusion criteria: Rajan et al. (2025)**, a case–control study of 34 intubated children aged 1–24 months with community-acquired severe pneumonia in Vellore, India, using multiplex PCR on tracheal aspirates, nasopharyngeal swabs, and bronchoalveolar lavage. It was appraised with the CCAT and scored **32/40 (80%)**, exceeding our pre-specified ≥75% inclusion threshold. The final synthesis therefore now includes **16 studies (8 risk factors, 8 aetiology)**.  **Impact on Results and Conclusions:** The update **reinforces rather than alters** our findings. Rajan et al. identified respiratory syncytial virus and *Streptococcus pneumoniae* as the predominant pathogens, with RSV significantly more frequent in cases than controls (OR 10.45, 95% CI 1.25–88.25) — directly consistent with our existing conclusion that viral pathogens predominate and RSV is the leading aetiological agent of childhood CAP in South Asia. No risk-factor studies were added, so the risk-factor synthesis is unchanged. Notably, this study strengthens our evidence base by sampling the **lower** respiratory tract, addressing a limitation of the predominantly nasopharyngeal sampling in the earlier studies. Our overall conclusions and recommendations therefore remain unchanged.  The manuscript, PRISMA flow diagram (Figure 1), Table 3, and Appendices S1, S2, and S4 have all been updated accordingly, with changes marked. |
| **A2** | Please amend your detailed Financial Disclosure statement. This is published with the article. It must therefore be completed in full sentences and contain the exact wording you wish to be published. If you did not receive any funding for this study, please simply state: “The authors received no specific funding for this work.” | We have amended the Financial Disclosure as: The authors received no specific funding for this work |
| **A3** | Please send a completed “Competing Interests” statement, including any COIs declared by your co-authors. If you have no competing interests to declare, please state “The authors have declared that no competing interests exist”. | We have added competing interests as: “The authors have declared that no competing interests exist”. |
| **A4** | Please remove the figure embedded in the manuscript as it is uploaded as a tif separately. | Figure 1: PRISMA flowchart has been removed from the manuscript, however, it uploaded as separate file. |
| **A5** | Figure resubmission: use PLOS’s NAAS tool to test the figure files and include the NAAS-processed figure files when submitting the revised manuscript. | We have checked the figure with PLOS’s NAAS tool and adjusted figure has been uploaded |
| **B. Reviewer #1 — Abstract** | | |
| **B1** | Please define CAP at its first occurrence. | We did it in the abstract. Community-acquired pneumonia (CAP) |
| **B2** | The abstract would benefit from a clearer statement of the motivation for conducting this systematic review. | We have amended the starting of the abstract to address the motivation as follows: “Pneumonia remains a leading cause of mortality among children under five years in South Asia, a region that, with sub-Saharan Africa, accounts for over half of the world's childhood pneumonia cases. To our knowledge, no prior review has jointly synthesised the risk factors and aetiological agents of community-acquired pneumonia (CAP) in under-fives specific to this region”. |
| **C. Reviewer #1 — Introduction** | | |
| **C1** | Haemophilus influenzae should be italicized. Please also check the manuscript for other scientific names and ensure that they are consistently italicized. | Throughought the manuscript we have corrected it. All scientific names are now italicised throughout the manuscript, tables and appendices (41 occurrences), including Haemophilus influenzae, Streptococcus pneumoniae, Staphylococcus aureus, Klebsiella pneumoniae, Mycoplasma pneumoniae, Mycobacterium tuberculosis and Escherichia coli, together with all abbreviated forms (e.g. S. pneumoniae, H. influenzae). Virus names have correctly been left in roman type. |
| **C2** | The statement that no systematic review has comprehensively examined both risk factors and aetiological agents of CAP among South Asian children under five is helpful, but it would be better to provide a brief discussion of the existing related systematic reviews. Specifically, the authors should summarize what these previous reviews covered and what they did not address. This would better justify the need for the present comprehensive systematic review and help readers understand how it adds to the existing literature. | We agree and have expanded the Introduction. We now summarise what the closest prior reviews covered and did not cover: von Mollendorf et al. synthesised aetiology across all LMICs, pooling South Asian data with other regions without disaggregation and not addressing risk factors; Soelaeman and Cahyadirga examined risk factors in selected Asian countries but not aetiology, with coverage extending beyond South Asia; and broader reviews of childhood pneumonia in developing countries (e.g. Rudan et al.; DeAntonio et al.) report risk factors and pathogens at global or multi-region level, not restricted to South Asia, to under-fives, or to community-acquired pneumonia. We now state explicitly that no existing review brings both bodies of evidence together for the South Asian under-five population or appraises study quality across them. We have also qualified our novelty claim with 'to our knowledge'. |
| **D. Reviewer #1 — Methods** | | |
| **D1** | Please clarify this exclusion criterion. Book chapters and conference proceedings should not be automatically classified as non-peer-reviewed, as some may undergo peer review depending on the publisher or conference. If these publication types were excluded regardless of peer-review status, please provide a clear rationale. | We accept this point and have reworded the criterion (See, Methods ; Eligibility Criteria, exclusion criterion 4), which previously implied these publication types are inherently non-peer-reviewed. The criterion now reads that studies were excluded if they were not full-text, peer-reviewed primary journal articles. We explicitly acknowledge that some book chapters and conference proceedings do undergo peer review, and give our rationale for excluding them regardless: they typically do not report the methodological detail (sampling, case definitions, analysis, effect estimates with confidence intervals) required for data extraction and for CCAT quality appraisal, and their peer-review status frequently cannot be reliably verified. We also confirm that no source was excluded on this basis alone that reported extractable primary data meeting all other criteria. |
| **D2** | Please clarify whether language restrictions were applied. If only English-language studies were included, this should be stated and justified due to possible selection bias. | Clarified (in the Methods, Eligibility Criteria (inclusion criterion 4); Discussion, Strengths and Limitations). English-language restriction was applied and was already listed among the inclusion criteria; we have now stated it explicitly and justified it (no translation resources were available to the review team). We have additionally added it to the Limitations, acknowledging potential language bias and noting that relevant studies in regional languages (e.g. Hindi, Bengali, Urdu, Nepali) may have been missed, while observing that the great majority of peer-reviewed research from the region is published in English and that no non-English record identified during screening met the other eligibility criteria. |
| **D3** | Please clarify whether the second reviewer was independent of the review team. | The second reviewer was independent of the review team and was not involved in the conduct of the review; screening was performed independently and blinded to the primary reviewer's decisions, and any disagreements were resolved by discussion until consensus was reached, with no third reviewer required. |
| **E. Reviewer #1 — Results and Discussion** | | |
| **E1** | The manuscript currently reads more like a literature review than a systematic review. Please clarify the novel contribution of the findings and strengthen the synthesis by identifying key patterns, gaps, and implications across studies. Since interventions may not be able to address all identified risk factors, it would be useful to highlight which factors are most influential or actionable. If possible, quantifying their relative influence would make the synthesis more informative. | We have substantially strengthened the synthesis (see, Results; synthesis passage following Table 2, Discussion). A new synthesis passage now moves beyond study-by-study description to identify three cross-cutting patterns: (i) risk factors differ in modifiability - young age was the most frequently reported association but is not itself actionable, and instead defines the 0-23-month window in which prevention must be concentrated, whereas malnutrition, incomplete immunisation and indoor smoke exposure are directly actionable and each carried a two- to four-fold increase in odds (underweight AOR 2.3, 95% CI 1.4-3.9; partial immunisation OR 2.41, 95% CI 1.06-5.49; indoor smoke-producing stoves OR 3.76, 95% CI 1.02-11.82; maternal smoking AOR 2.5, 95% CI 1.1-5.7); (ii) when ranked by magnitude rather than by the number of studies reporting them, household air pollution carried effect sizes at least as large as the nutritional and immunisation factors that dominate current policy attention, identifying it as an under-prioritised target; and (iii) protective exposures are equally actionable, with adequate cross-ventilation (AOR 0.58, 95% CI 0.40-0.83) and maternal knowledge of pneumonia symptoms (OR 0.36, 95% CI 0.26-0.48) approximately halving the odds. We conclude that the evidence points to a small number of high-yield, co-occurring targets clustered in the first two years of life rather than an undifferentiated list of risks.  We have deliberately refrained from formally pooling or numerically ranking these estimates, and state explicitly that they derive from studies with heterogeneous designs, exposure definitions and confounder adjustment and are therefore not formally comparable; they are presented to indicate relative magnitude and consistency of direction only. We have also added the key evidence gaps: no included study quantified the interaction between undernutrition and household air pollution despite their frequent co-occurrence; no eligible study was identified from Sri Lanka, Afghanistan or the Maldives; and population-based aetiological data outside hospital settings remain scarce. |
| **E2** | It would also be helpful to relate the findings to regional intervention strategies and surveillance needs. In particular, the authors should discuss how the identified aetiological agents could inform targeted prevention, diagnostic priorities, and surveillance monitoring for community-acquired pneumonia among under-five children in South Asia. | We have added (see, Discussion, Implications for Policy and Practice) a new Discussion passage translating the aetiological findings into three regional priorities. (1) Targeted prevention: the consistent predominance of RSV (leading pathogen in seven of eight aetiological studies; mean detection 27.5%; significant case-control association in the newest evidence) makes RSV-directed prophylaxis the highest-yield addition to the preventive portfolio, since maternal RSV vaccination and long-acting monoclonals (e.g. nirsevimab) protect precisely the 0-23-month window this review identifies as peak risk; in parallel, the persistence of Streptococcus pneumoniae in a region with incomplete PCV coverage (only 28% of cases PCV-vaccinated in the most recent study) argues for accelerating PCV coverage with serotype-replacement monitoring. (2) Diagnostic priorities: the low yield of blood culture (positive in under 9% of severe cases) confirms that culture-based diagnosis underestimates bacterial contribution, supporting investment in affordable multiplex molecular platforms and in specimen types representative of the lower respiratory tract; we also caution that, because potential pathogens are detected in the upper airway of children without pneumonia, nasopharyngeal positivity alone must not be equated with causation. (3) Surveillance: the region lacks standardised, population-based pneumonia aetiology surveillance, and we recommend sentinel sites applying uniform WHO case definitions, collecting paired upper and lower respiratory specimens, and reporting RSV and pneumococcal detection alongside vaccination status, so that the impact of PCV and forthcoming RSV interventions can be tracked. |

We hope the revisions are satisfactory and that the manuscript is now suitable for publication in PLOS Global Public Health. Thank you again for your time and valuable feedback.

Yours sincerely,

**Md. Mamunur Rashid Sheikh, PhD, FHEA**

Corresponding author, on behalf of all co-authors

Email: mamunrashid@sw.jnu.ac.bd
