# Supplementary material for: Risk factors and aetiological agents of community-acquired pneumonia in children under five years in South Asia: A systematic review
Source: PLOS Glob Public Health. 2026 Nov 10;6(11):e0006388. doi: 10.1371/journal.pgph.0006388 (PMC13750549; doi:10.1371/journal.pgph.0006388)
Supplement: Supplementary file 7 [file pgph.0006388.s009.docx]

**Response to the Editor and Reviewer Comments**

**Manuscript ID: PGPH-D-26-01392R1**

Dear Dr. Zimmerman,

We thank you and the two reviewers for the constructive feedback of our manuscript. We are pleased that both reviewers confirm all previous comments have been addressed and rate the manuscript favourably on every publication-criteria question. Reviewer #2 raised several further points to strengthen the discussion of our findings and identified specific formatting errors, all of which we have addressed below and in the revised manuscript. We have also reviewed the reference list per the Journal Requirements and corrected several errors we identified during that process. Our point-by-point response follows, with reviewer comments reproduced in italics and our responses in bold-labelled paragraphs beneath each.

## **Journal Requirements**

**1. Recommendation to cite specific previously published works**

*“If the reviewer comments include a recommendation to cite specific previously published works, please review and evaluate these publications to determine whether they are relevant...”*

**Response:** Neither reviewer recommended specific additional works to cite in this round. No action was required on this point.

**2. Completeness and correctness of the reference list**

*“Please review your reference list to ensure that it is complete and correct... Any changes to the reference list should be mentioned in the rebuttal letter...”*

**Response:** We reviewed the full reference list (65 entries) against the in-text citations and identified three references that had been inadvertently duplicated under different numbers: reference 27 duplicated reference 24 (Soelaeman & Cahyadirga), reference 32 duplicated reference 25 (Jullien et al.), and reference 36 duplicated reference 33 (Goyal et al.). We have removed the duplicate entry in each pair, renumbered the reference list sequentially (now 62 entries), and updated every corresponding in-text citation number throughout the manuscript, tables, and figure legends. We also confirmed that no reference in the list has been retracted; none required a retraction notice or removal.

**3. Independent verification of reference authenticity and claim accuracy**

Beyond the duplicate-reference check above, and as a final check before resubmission, we carried out a full, independent verification of all 62 references for authenticity and for accurate support of the claims attributed to them in the text. This identified a small number of references where the citation did not fully match the claim it was supporting, or where a figure quoted from the source had been transcribed slightly inaccurately. We have corrected each of these as further tracked changes in the revised manuscript, including replacing a few references with more suitable sources and correcting several numerical values to match the cited studies.

## **Reviewer #1**

*“The authors substantially improved the manuscript and sufficiently addressed my comments. I have no further comments.”*

**Response:** We thank Reviewer #1 for their time and positive assessment across both rounds of review. No further changes were required in response to Reviewer #1.

## **Reviewer #2**

We thank Reviewer #2 for describing our methodology as robust and for the detailed, constructive comments below, each of which we address in turn.

**1. Only a 10% random sample of screening decisions was independently validated**

*“The methodology is robust though there is only a 10% random selection of articles validated to reduce selection bias.”*

**Response:** We agree this residual limitation warranted more explicit discussion. We have expanded the relevant limitation in the Strengths and Limitations subsection of the Discussion to read: *“Although a random 10% sample of full-text screening decisions was independently cross-checked by a second, blinded reviewer, with disagreements resolved by consensus, the review was otherwise conducted by a single primary reviewer for an academic dissertation. Full independent dual screening of all records was not feasible within the scope of this dissertation-based review, and the extent and direction of any residual selection bias arising from the remaining unchecked decisions cannot be determined. We recommend that future updates of this review adopt full dual independent screening to minimise this risk.”*

**2. Changes in aetiology over time, spanning the roll-out of vaccination programmes, were not explored**

*“For example the time frame of articles covers the implementation of vaccination programmes, and the relevant changes over time in aetiology are not explored.”*

**Response:** This is a valuable point. We have added the following to the Limitations subsection, immediately following our existing statement on the 24-year study period: *“The included studies span a period during which conjugate vaccination programmes against Haemophilus influenzae type b and Streptococcus pneumoniae were progressively introduced across South Asian countries, alongside evolving healthcare systems and socioeconomic conditions. The majority of the aetiological studies included in this review were conducted before, or during the early phase of, national pneumococcal conjugate vaccine (PCV) introduction in their respective countries; consequently, the pooled aetiological profile reported here should be interpreted as a time-averaged snapshot rather than the current picture, and may overstate the contemporary burden of vaccine-preventable bacterial pathogens relative to settings with mature PCV programmes.”*

**3. Cross-country variation in healthcare systems, and the absence of Sri Lankan data, were not explored**

*“Additionally the significant variation in healthcare systems and impacts on variations between countries in S Asia are not explored with notable lack of data from Sri Lanka which has low pneumonia mortality rates.”*

**Response:** We agree this is a notable gap. Although Sri Lanka, Afghanistan, and the Maldives were within the scope of our eligibility criteria, no studies from these three countries met full eligibility, and none were included in the final synthesis. We have added the following new point to the Limitations subsection: *“Although Sri Lanka, Afghanistan, and the Maldives were eligible under our inclusion criteria, no studies from these countries met the full eligibility criteria and none were included in the final synthesis. This is a notable gap, particularly for Sri Lanka, which has achieved comparatively low under-five pneumonia mortality relative to other countries in the region, an outcome attributed in the wider literature to its universal, decentralised primary healthcare system. The absence of Sri Lankan data means this review cannot examine how cross-country variation in health-system organisation and access may modify the risk factors and aetiological patterns identified here. We recommend that future primary research and reviews specifically target Sri Lanka, Afghanistan, and the Maldives, both to fill this evidence gap and to enable comparison between low- and higher-mortality health systems within South Asia.”*

**4. Line 178: reference present but using a different format, and/or numbered incorrectly (28)**

*“Line 178, reference present but using different format and if not, numbered incorrectly (28)”*

**Response:** We identified the source of this error: the sentence introducing our PRISMA methodology mixed author-name citation style with numbered-citation style and contained an unbalanced parenthesis (“...reporting (Moher et al., (28).”). We have corrected this to read “...reporting (28).”, consistent with the numbered citation style used throughout the rest of the manuscript. As noted above, this citation number has also been checked against the corrected, renumbered reference list.

**5. Line 319: reference misformatted**

*“Line 319, reference misformatted”*

**Response:** We reviewed the citation at this location against the numbered-citation style used throughout the manuscript and corrected its formatting to match. This citation has also been renumbered where necessary following removal of the duplicate reference entries described under Journal Requirements above.

**6. Line 601: inconsistent use of italics for pathogen names**

*“Line 601 inconsistent use of italics for pathogen names”*

**Response:** We identified two locations where genus and species names were not italicised: in the Introduction (“S. pneumoniae and Staphylococcus aureus”) and in the Discussion (“Streptococcus pneumoniae”, Implications for Policy and Practice). Both have been corrected to italics. We additionally re-checked every instance of the genus names Streptococcus, Staphylococcus, Haemophilus, Klebsiella, Pseudomonas, Escherichia, Moraxella, Salmonella, Serratia, Bordetella, and Acinetobacter throughout the manuscript text, tables, and figure legends to confirm consistent italicisation.

**7. Publication of peer review history**

*Reviewer #1: No. Reviewer #2: Yes (J N Periselneris).*

**Response:** Noted; no action required from the authors on this point.

## **Additional corrections identified during this revision**

While addressing the points above, we also identified and corrected the following minor errors, which we mention here for completeness though they were not raised by the editor or reviewers:

• The Limitations subsection had inadvertently raised the same point twice — the restriction to English-language publications was stated as both the first and (in near-identical wording) the fourth limitation. We have merged these into a single point.

• Two stray duplicated periods (“First, .The search...” and “...treatment decisions. . The aetiological findings...”) have been removed.

• Inconsistent spacing after the reference number in entries 1 and 3 of the reference list has been standardised to match the rest of the list.

• A stray closing parenthesis following the ORCID URL has been corrected in both authors' affiliation lines.

We believe these revisions fully address the points raised by the editor and both reviewers. We thank you again for the opportunity to revise our manuscript and look forward to your response.

Yours sincerely, on behalf of all authors,

**Md. Mamunur Rashid Sheikh (Corresponding Author)**
