# Supplementary material for: Risk factors and aetiological agents of community-acquired pneumonia in children under five years in South Asia: A systematic review
Source: PLOS Glob Public Health. 2026 Nov 10;6(11):e0006388. doi: 10.1371/journal.pgph.0006388 (PMC13750549; doi:10.1371/journal.pgph.0006388)
Supplement: Peer review history

# Peer review: Decision Letter 0

**DOI:** 10.1371/journal.pgph.0006388.r001

**Authors:** Gemma Lea Saravanos

29 Jun 2026

PGPH-D-26-01392

Risk Factors and Aetiological Agents of Community-Acquired Pneumonia in Children Under Five Years in South Asia: A Systematic Review

PLOS Global Public Health

Dear Dr. Sheikh,

Thank you for submitting your manuscript to PLOS Global Public Health. After careful consideration, we feel that it has merit but does not fully meet PLOS Global Public Health’s publication criteria as it currently stands and it requires substantial revisions before we can consider publication.

Most importantly, the literature search is now 2 years old and this should be updated to ensure that the review provides an up-to-date overview of the research topic covered. Additionally, the provided peer review comments should be addressed.

Please refer to our author guidelines for systematic reviews/meta-analyses to guide any future revisions.

Please submit your revised manuscript by Aug 28 2026 11:59PM. If you will need more time than this to complete your revisions, please reply to this message or contact the journal office at globalpubhealth@plos.org. When you're ready to submit your revision, log on to https://www.editorialmanager.com/pgph/ and select the 'Submissions Needing Revision' folder to locate your manuscript file.

Please include the following items when submitting your revised manuscript:

- A letter that responds to each point raised by the editor and reviewer(s). You should upload this letter as a separate file labeled 'Response to Reviewers'.
- A marked-up copy of your manuscript that highlights changes made to the original version. You should upload this as a separate file labeled 'Revised Manuscript with Track Changes'.
- An unmarked version of your revised paper without tracked changes. You should upload this as a separate file labeled 'Manuscript'.

Guidelines for resubmitting your figure files are available below the reviewer comments at the end of this letter.

As the corresponding author, your ORCID iD is verified in the submission system and will appear in the published article. PLOS supports the use of ORCID, and we encourage all coauthors to register for an ORCID iD and use it as well. Please encourage your coauthors to verify their ORCID iD within the submission system before final acceptance, as unverified ORCID iDs will not appear in the published article. *Only*  the individual author can complete the verification step; PLOS staff *cannot*  verify ORCID iDs on behalf of authors.

Yours Sincerely,

Dr Gemma L Saravanos

Academic Editor

PLOS Global Public Health

==============================

Journal Requirements:

1. We note that the most recent literature search was completed over a year before the manuscript was submitted to the journal. Systematic and scoping reviews should provide an up-to-date overview of the research topic covered. Therefore, we request that you please update your searches as part of your revision to ensure that the most up-to-date literature is included in your review, or provide scientific justification for not doing so. Please ensure you mention this in your response to reviewers, and discuss whether this has any impact on the Results and Conclusions of the study. If you are submitting a protocol, it is only necessary to state whether the searches will be updated prior to the publication of the results.

1. Please amend your detailed Financial Disclosure statement. This is published with the article. It must therefore be completed in full sentences and contain the exact wording you wish to be published.
  If you did not receive any funding for this study, please simply state: “The authors received no specific funding for this work.”

1. Please send a completed 'Competing Interests' statement, including any COIs declared by your co-authors. If you have no competing interests to declare, please state "The authors have declared that no competing interests exist".

1. Please remove the figure embedded in the manuscript as it is upload as tif separately.

If the reviewer comments include a recommendation to cite specific previously published works, please review and evaluate these publications to determine whether they are relevant and should be cited. There is no requirement to cite these works unless the editor has indicated otherwise.

[Note: HTML markup is below. Please do not edit.]

Reviewers' comments:

Reviewer's Responses to Questions

**Comments to the Author**

1. Does this manuscript meet PLOS Global Public Health’s publication criteria?

Reviewer #1: Partly

**********

2. Has the statistical analysis been performed appropriately and rigorously?

Reviewer #1: N/A

**********

3. Have the authors made all data underlying the findings in their manuscript fully available (please refer to the Data Availability Statement at the start of the manuscript PDF file)?

The PLOS Data policy

Reviewer #1: Yes

**********

4. Is the manuscript presented in an intelligible fashion and written in standard English?

Reviewer #1: Yes

**********

Reviewer #1: The study provides some useful insights on the risk factors and aetiological agents associated with community-acquired pneumonia (CAP) among children under five in South Asia. However, I am not yet fully convinced about the necessity and novelty of conducting this as a systematic review. The manuscript currently reads more like a structured literature review, and the added value of the systematic approach should be made clearer. The topic has potential for publication, particularly for a regionally focused journal, but for a broader global health journal, the authors should better articulate the wider relevance, novelty, and implications of the findings. Below are some of my comments for consideration.

Abstract

1. Please define CAP at its first occurrence.

2. The abstract would benefit from a clearer statement of the motivation for conducting this systematic review.

Introduction

1. Haemophilus influenzae should be italicized. Please also check the manuscript for other scientific names and ensure that they are consistently italicized.

2. The statement that no systematic review has comprehensively examined both risk factors and aetiological agents of CAP among South Asian children under five is helpful, but it would be better to provide a brief discussion of the existing related systematic reviews. Specifically, the authors should summarize what these previous reviews covered and what they did not address. This would better justify the need for the present comprehensive systematic review and help readers understand how it adds to the existing literature.

Methods

1. Please clarify this exclusion criterion. Book chapters and conference proceedings should not be automatically classified as non-peer-reviewed, as some may undergo peer review depending on the publisher or conference. If these publication types were excluded regardless of peer-review status, please provide a clear rationale.

2. Please clarify whether language restrictions were applied. If only English-language studies were included, this should be stated and justified due to possible selection bias.

3. Please clarify whether the second reviewer was independent of the review team.

Results and Discussion

1. The manuscript currently reads more like a literature review than a systematic review. Please clarify the novel contribution of the findings and strengthen the synthesis by identifying key patterns, gaps, and implications across studies. Since interventions may not be able to address all identified risk factors, it would be useful to highlight which factors are most influential or actionable. If possible, quantifying their relative influence would make the synthesis more informative.

2. It would also be helpful to relate the findings to regional intervention strategies and surveillance needs. In particular, the authors should discuss how the identified aetiological agents could inform targeted prevention, diagnostic priorities, and surveillance monitoring for community-acquired pneumonia among under-five children in South Asia.

**********

what does this mean?). If published, this will include your full peer review and any attached files.

**Do you want your identity to be public for this peer review?**  If you choose “no”, your identity will remain anonymous but your review may still be made public.

For information about this choice, including consent withdrawal, please see our Privacy Policy

Reviewer #1: No

**********

[NOTE: If reviewer comments were submitted as an attachment file, they will be attached to this email and accessible via the submission site. Please log into your account, locate the manuscript record, and check for the action link "View Attachments". If this link does not appear, there are no attachment files.]

# Author comment: Author response to Decision Letter 1

**DOI:** 10.1371/journal.pgph.0006388.r002

14 Jul 2026

Dear Dr Saravanos,

Thank you for the opportunity to revise our manuscript. We are grateful to you and the reviewer for the constructive comments, which have helped us improve the manuscript. Our point-by-point responses are provided in the table below. All corresponding changes are highlighted in the “Revised Manuscript with Track Changes” file.

A. Journal Requirements

Comment A1:

We note that the most recent literature search was completed over a year before the manuscript was submitted to the journal. Systematic and scoping reviews should provide an up-to-date overview of the research topic covered. Therefore, we request that you please update your searches as part of your revision to ensure that the most up-to-date literature is included in your review, or provide scientific justification for not doing so. Please ensure you mention this in your response to reviewers, and discuss whether this has any impact on the Results and Conclusions of the study.

Response:

We thank the Editor for this important observation and fully agree that a systematic review should reflect the most current evidence. We have therefore updated our search rather than seeking a justification for not doing so.

Using the identical search strategy, MeSH terms, and eligibility criteria as the original search, we re-ran the search across all four databases (MEDLINE, PubMed, Global Health, and Web of Science) to cover the period August 2024 to January 2026. This identified 204 additional records (Web of Science = 13, PubMed = 75, MEDLINE = 54, Global Health = 62). After removing 147 duplicates, 57 records were screened, of which 49 were excluded at title and abstract. Of the 8 full-text articles assessed, 7 were excluded (3 without separate under-five data; 2 without pneumonia-specific data; 2 reporting different outcomes).

One study met all inclusion criteria: Rajan et al. (2025), a case–control study of 34 intubated children aged 1–24 months with community-acquired severe pneumonia in Vellore, India, using multiplex PCR on tracheal aspirates, nasopharyngeal swabs, and bronchoalveolar lavage. It was appraised with the CCAT and scored 32/40 (80%), exceeding our pre-specified ≥75% inclusion threshold. The final synthesis therefore now includes 16 studies (8 risk factors, 8 aetiology).

Impact on Results and Conclusions: The update reinforces rather than alters our findings. Rajan et al. identified respiratory syncytial virus and Streptococcus pneumoniae as the predominant pathogens, with RSV significantly more frequent in cases than controls (OR 10.45, 95% CI 1.25–88.25) — directly consistent with our existing conclusion that viral pathogens predominate and RSV is the leading aetiological agent of childhood CAP in South Asia. No risk-factor studies were added, so the risk-factor synthesis is unchanged. Notably, this study strengthens our evidence base by sampling the lower respiratory tract, addressing a limitation of the predominantly nasopharyngeal sampling in the earlier studies. Our overall conclusions and recommendations therefore remain unchanged.

The manuscript, PRISMA flow diagram (Figure 1), Table 3, and Appendices S1, S2, and S4 have all been updated accordingly, with changes marked.

Comment A2:

Please amend your detailed Financial Disclosure statement. This is published with the article. It must therefore be completed in full sentences and contain the exact wording you wish to be published. If you did not receive any funding for this study, please simply state: “The authors received no specific funding for this work.”

Response:

We have amended the Financial Disclosure as: The authors received no specific funding for this work

Comment A3:

Please send a completed “Competing Interests” statement, including any COIs declared by your co-authors. If you have no competing interests to declare, please state “The authors have declared that no competing interests exist”.

Response:

We have added competing interests as: “The authors have declared that no competing interests exist”.

Comment A4:

Please remove the figure embedded in the manuscript as it is uploaded as a tif separately.

Response:

Figure 1: PRISMA flowchart has been removed from the manuscript, however, it uploaded as separate file.

Comment A5:

Figure resubmission: use PLOS’s NAAS tool to test the figure files and include the NAAS-processed figure files when submitting the revised manuscript.

Response:

We have checked the figure with PLOS’s NAAS tool and adjusted figure has been uploaded

B. Reviewer #1 — Abstract

Comment B1:

Please define CAP at its first occurrence.

Response:

We did it in the abstract. Community-acquired pneumonia (CAP)

Comment B2:

The abstract would benefit from a clearer statement of the motivation for conducting this systematic review.

Response:

We have amended the starting of the abstract to address the motivation as follows: “Pneumonia remains a leading cause of mortality among children under five years in South Asia, a region that, with sub-Saharan Africa, accounts for over half of the world's childhood pneumonia cases. To our knowledge, no prior review has jointly synthesised the risk factors and aetiological agents of community-acquired pneumonia (CAP) in under-fives specific to this region”.

C. Reviewer #1 — Introduction

Comment C1:

Haemophilus influenzae should be italicized. Please also check the manuscript for other scientific names and ensure that they are consistently italicized.

Response:

Throughought the manuscript we have corrected it. All scientific names are now italicised throughout the manuscript, tables and appendices (41 occurrences), including Haemophilus influenzae, Streptococcus pneumoniae, Staphylococcus aureus, Klebsiella pneumoniae, Mycoplasma pneumoniae, Mycobacterium tuberculosis and Escherichia coli, together with all abbreviated forms (e.g. S. pneumoniae, H. influenzae). Virus names have correctly been left in roman type.

Comment C2:

The statement that no systematic review has comprehensively examined both risk factors and aetiological agents of CAP among South Asian children under five is helpful, but it would be better to provide a brief discussion of the existing related systematic reviews. Specifically, the authors should summarize what these previous reviews covered and what they did not address. This would better justify the need for the present comprehensive systematic review and help readers understand how it adds to the existing literature.

Response:

We agree and have expanded the Introduction. We now summarise what the closest prior reviews covered and did not cover: von Mollendorf et al. synthesised aetiology across all LMICs, pooling South Asian data with other regions without disaggregation and not addressing risk factors; Soelaeman and Cahyadirga examined risk factors in selected Asian countries but not aetiology, with coverage extending beyond South Asia; and broader reviews of childhood pneumonia in developing countries (e.g. Rudan et al.; DeAntonio et al.) report risk factors and pathogens at global or multi-region level, not restricted to South Asia, to under-fives, or to community-acquired pneumonia. We now state explicitly that no existing review brings both bodies of evidence together for the South Asian under-five population or appraises study quality across them. We have also qualified our novelty claim with 'to our knowledge'.

D. Reviewer #1 — Methods

Comment D1:

Please clarify this exclusion criterion. Book chapters and conference proceedings should not be automatically classified as non-peer-reviewed, as some may undergo peer review depending on the publisher or conference. If these publication types were excluded regardless of peer-review status, please provide a clear rationale.

Response:

We accept this point and have reworded the criterion (See, Methods ; Eligibility Criteria, exclusion criterion 4), which previously implied these publication types are inherently non-peer-reviewed. The criterion now reads that studies were excluded if they were not full-text, peer-reviewed primary journal articles. We explicitly acknowledge that some book chapters and conference proceedings do undergo peer review, and give our rationale for excluding them regardless: they typically do not report the methodological detail (sampling, case definitions, analysis, effect estimates with confidence intervals) required for data extraction and for CCAT quality appraisal, and their peer-review status frequently cannot be reliably verified. We also confirm that no source was excluded on this basis alone that reported extractable primary data meeting all other criteria.

Comment D2:

Please clarify whether language restrictions were applied. If only English-language studies were included, this should be stated and justified due to possible selection bias.

Response:

Clarified (in the Methods, Eligibility Criteria (inclusion criterion 4); Discussion, Strengths and Limitations). English-language restriction was applied and was already listed among the inclusion criteria; we have now stated it explicitly and justified it (no translation resources were available to the review team). We have additionally added it to the Limitations, acknowledging potential language bias and noting that relevant studies in regional languages (e.g. Hindi, Bengali, Urdu, Nepali) may have been missed, while observing that the great majority of peer-reviewed research from the region is published in English and that no non-English record identified during screening met the other eligibility criteria.

Comment D3:

Please clarify whether the second reviewer was independent of the review team.

Response:

The second reviewer was independent of the review team and was not involved in the conduct of the review; screening was performed independently and blinded to the primary reviewer's decisions, and any disagreements were resolved by discussion until consensus was reached, with no third reviewer required.

E. Reviewer #1 — Results and Discussion

Comment E1:

The manuscript currently reads more like a literature review than a systematic review. Please clarify the novel contribution of the findings and strengthen the synthesis by identifying key patterns, gaps, and implications across studies. Since interventions may not be able to address all identified risk factors, it would be useful to highlight which factors are most influential or actionable. If possible, quantifying their relative influence would make the synthesis more informative.

Response:

We have substantially strengthened the synthesis (see, Results; synthesis passage following Table 2, Discussion). A new synthesis passage now moves beyond study-by-study description to identify three cross-cutting patterns: (i) risk factors differ in modifiability - young age was the most frequently reported association but is not itself actionable, and instead defines the 0-23-month window in which prevention must be concentrated, whereas malnutrition, incomplete immunisation and indoor smoke exposure are directly actionable and each carried a two- to four-fold increase in odds (underweight AOR 2.3, 95% CI 1.4-3.9; partial immunisation OR 2.41, 95% CI 1.06-5.49; indoor smoke-producing stoves OR 3.76, 95% CI 1.02-11.82; maternal smoking AOR 2.5, 95% CI 1.1-5.7); (ii) when ranked by magnitude rather than by the number of studies reporting them, household air pollution carried effect sizes at least as large as the nutritional and immunisation factors that dominate current policy attention, identifying it as an under-prioritised target; and (iii) protective exposures are equally actionable, with adequate cross-ventilation (AOR 0.58, 95% CI 0.40-0.83) and maternal knowledge of pneumonia symptoms (OR 0.36, 95% CI 0.26-0.48) approximately halving the odds. We conclude that the evidence points to a small number of high-yield, co-occurring targets clustered in the first two years of life rather than an undifferentiated list of risks.

We have deliberately refrained from formally pooling or numerically ranking these estimates, and state explicitly that they derive from studies with heterogeneous designs, exposure definitions and confounder adjustment and are therefore not formally comparable; they are presented to indicate relative magnitude and consistency of direction only. We have also added the key evidence gaps: no included study quantified the interaction between undernutrition and household air pollution despite their frequent co-occurrence; no eligible study was identified from Sri Lanka, Afghanistan or the Maldives; and population-based aetiological data outside hospital settings remain scarce.

Comment E2:

It would also be helpful to relate the findings to regional intervention strategies and surveillance needs. In particular, the authors should discuss how the identified aetiological agents could inform targeted prevention, diagnostic priorities, and surveillance monitoring for community-acquired pneumonia among under-five children in South Asia.

Response:

We have added (see, Discussion, Implications for Policy and Practice) a new Discussion passage translating the aetiological findings into three regional priorities. (1) Targeted prevention: the consistent predominance of RSV (leading pathogen in seven of eight aetiological studies; mean detection 27.5%; significant case-control association in the newest evidence) makes RSV-directed prophylaxis the highest-yield addition to the preventive portfolio, since maternal RSV vaccination and long-acting monoclonals (e.g. nirsevimab) protect precisely the 0-23-month window this review identifies as peak risk; in parallel, the persistence of Streptococcus pneumoniae in a region with incomplete PCV coverage (only 28% of cases PCV-vaccinated in the most recent study) argues for accelerating PCV coverage with serotype-replacement monitoring. (2) Diagnostic priorities: the low yield of blood culture (positive in under 9% of severe cases) confirms that culture-based diagnosis underestimates bacterial contribution, supporting investment in affordable multiplex molecular platforms and in specimen types representative of the lower respiratory tract; we also caution that, because potential pathogens are detected in the upper airway of children without pneumonia, nasopharyngeal positivity alone must not be equated with causation. (3) Surveillance: the region lacks standardised, population-based pneumonia aetiology surveillance, and we recommend sentinel sites applying uniform WHO case definitions, collecting paired upper and lower respiratory specimens, and reporting RSV and pneumococcal detection alongside vaccination status, so that the impact of PCV and forthcoming RSV interventions can be tracked.

We hope the revisions are satisfactory and that the manuscript is now suitable for publication in PLOS Global Public Health. Thank you again for your time and valuable feedback.

Yours sincerely,

Md. Mamunur Rashid Sheikh, PhD, FHEA

Corresponding author, on behalf of all co-authors

Email: mamunrashid@sw.jnu.ac.bd

Attachment Submitted filename: Response_to_Reviewers_Table (CAP).docx

# Peer review: Decision Letter 1

**DOI:** 10.1371/journal.pgph.0006388.r003

**Authors:** Steve Zimmerman

1 Sep 2026

PGPH-D-26-01392R1

Risk Factors and Aetiological Agents of Community-Acquired Pneumonia in Children Under Five Years in South Asia: A Systematic Review

PLOS Global Public Health

Dear Dr. Sheikh,

Thank you for submitting your manuscript to PLOS Global Public Health. After careful consideration, we feel that it has merit but does not fully meet PLOS Global Public Health’s publication criteria as it currently stands. Therefore, we invite you to submit a revised version of the manuscript that addresses the points raised during the review process.

The manuscript has been evaluated by two reviewers, and their comments are available below.

Could you please revise the manuscript to carefully address the concerns raised?

Please submit your revised manuscript by Oct 01 2026 11:59PM. If you will need more time than this to complete your revisions, please reply to this message or contact the journal office at globalpubhealth@plos.org. When you're ready to submit your revision, log on to https://www.editorialmanager.com/pgph/ and select the 'Submissions Needing Revision' folder to locate your manuscript file.

Please include the following items when submitting your revised manuscript:

- A letter that responds to each point raised by the editor and reviewer(s). You should upload this letter as a separate file labeled 'Response to Reviewers'.
- A marked-up copy of your manuscript that highlights changes made to the original version. You should upload this as a separate file labeled 'Revised Manuscript with Track Changes'.
- An unmarked version of your revised paper without tracked changes. You should upload this as a separate file labeled 'Manuscript'.

Guidelines for resubmitting your figure files are available below the reviewer comments at the end of this letter.

As the corresponding author, your ORCID iD is verified in the submission system and will appear in the published article. PLOS supports the use of ORCID, and we encourage all coauthors to register for an ORCID iD and use it as well. Please encourage your coauthors to verify their ORCID iD within the submission system before final acceptance, as unverified ORCID iDs will not appear in the published article. *Only*  the individual author can complete the verification step; PLOS staff *cannot*  verify ORCID iDs on behalf of authors.

We look forward to receiving your revised manuscript.

Kind regards,

Steve Zimmerman, PhD

PLOS Staff Editor

**Journal Requirements:**

If the reviewer comments include a recommendation to cite specific previously published works, please review and evaluate these publications to determine whether they are relevant and should be cited. There is no requirement to cite these works unless the editor has indicated otherwise.

Please review your reference list to ensure that it is complete and correct. If you have cited papers that have been retracted, please include the rationale for doing so in the manuscript text, or remove these references and replace them with relevant current references. Any changes to the reference list should be mentioned in the rebuttal letter that accompanies your revised manuscript. If you need to cite a retracted article, indicate the article’s retracted status in the References list and also include a citation and full reference for the retraction notice.

**Additional Editor Comments (if provided):**

[Note: HTML markup is below. Please do not edit.]

Reviewers' comments:

Reviewer's Responses to Questions

**Comments to the Author**

Reviewer #1: All comments have been addressed

Reviewer #2: All comments have been addressed

**********

publication criteria?

Reviewer #1: Yes

Reviewer #2: Yes

**********

3. Has the statistical analysis been performed appropriately and rigorously?

Reviewer #1: Yes

Reviewer #2: Yes

**********

4. Have the authors made all data underlying the findings in their manuscript fully available (please refer to the Data Availability Statement at the start of the manuscript PDF file)?

The PLOS Data policy

Reviewer #1: Yes

Reviewer #2: Yes

**********

5. Is the manuscript presented in an intelligible fashion and written in standard English?

Reviewer #1: Yes

Reviewer #2: Yes

**********

**Reviewer #1:**  The authors substantially improved the manuscript and sufficiently addressed my comments. I have no further comments.

**Reviewer #2** : This is a well written meta-analysis synthesising epidemiological and pathogen risk factors for community acquired pneumonia in the under 5s in South Asia. The methodology is robust though there is only a 10% random selection of articles validated to reduce selection bias.

While there is a thorough description of limitations, this could be discussed further, particularly implications of conclusions. For example the time frame of articles covers the implementation of vaccination programmes, and the relevant changes over time in aetiology are not explored. Additionally the significant variation in healthcare systems and impacts on variations between countries in S Asia are not explored with notable lack of data from Sri Lanka which has low pneumonia mortality rates.

There are some formatting issues, e.g.

Line 178, reference present but using different format and if not, numbered incorrectly (28)

Line 319, reference misformatted

Line 601 inconsistent use of italics for pathogen names

**********

what does this mean?). If published, this will include your full peer review and any attached files.

**Do you want your identity to be public for this peer review?**  If you choose “no”, your identity will remain anonymous but your review may still be made public.

For information about this choice, including consent withdrawal, please see our Privacy Policy

Reviewer #1: No

Reviewer #2: **Yes:**  J N Periselneris

**********

[NOTE: If reviewer comments were submitted as an attachment file, they will be attached to this email and accessible via the submission site. Please log into your account, locate the manuscript record, and check for the action link "View Attachments". If this link does not appear, there are no attachment files.]

**Figure Resubmissions:**

# Author comment: Author response to Decision Letter 2

**DOI:** 10.1371/journal.pgph.0006388.r004

7 Sep 2026

Attachment Submitted filename: Response to Reviewers - R2.docx

# Peer review: Decision Letter 2

**DOI:** 10.1371/journal.pgph.0006388.r005

**Authors:** Priyanka Roy

28 Sep 2026

Risk Factors and Aetiological Agents of Community-Acquired Pneumonia in Children Under Five Years in South Asia: A Systematic Review

PGPH-D-26-01392R2

Dear Dr Sheikh,

We are pleased to inform you that your manuscript 'Risk Factors and Aetiological Agents of Community-Acquired Pneumonia in Children Under Five Years in South Asia: A Systematic Review' has been provisionally accepted for publication in PLOS Global Public Health.

Before your manuscript can be formally accepted you will need to complete some formatting changes, which you will receive in a follow up email. A member of our team will be in touch with a set of requests.

Please note that your manuscript will not be scheduled for publication until you have made the required changes, so a swift response is appreciated.

IMPORTANT: The editorial review process is now complete. PLOS will only permit corrections to spelling, formatting or significant scientific errors from this point onwards. Requests for major changes, or any which affect the scientific understanding of your work, will cause delays to the publication date of your manuscript.

If your institution or institutions have a press office, please notify them about your upcoming paper to help maximize its impact. If they'll be preparing press materials, please inform our press team as soon as possible -- no later than 48 hours after receiving the formal acceptance. Your manuscript will remain under strict press embargo until 2 pm Eastern Time on the date of publication. For more information, please contact globalpubhealth@plos.org.

Thank you again for supporting Open Access publishing; we are looking forward to publishing your work in PLOS Global Public Health.

Best regards,

Priyanka Roy, MD, MPH, PhD

Academic Editor

PLOS Global Public Health

***********************************************************

Reviewer Comments (if any, and for reference):

Reviewer's Responses to Questions

**Comments to the Author**

Reviewer #1: All comments have been addressed

Reviewer #2: All comments have been addressed

**********

publication criteria?

Reviewer #1: Yes

Reviewer #2: Yes

**********

3. Has the statistical analysis been performed appropriately and rigorously?

Reviewer #1: Yes

Reviewer #2: N/A

**********

4. Have the authors made all data underlying the findings in their manuscript fully available (please refer to the Data Availability Statement at the start of the manuscript PDF file)?

The PLOS Data policy

Reviewer #1: Yes

Reviewer #2: Yes

**********

5. Is the manuscript presented in an intelligible fashion and written in standard English?

Reviewer #1: Yes

Reviewer #2: Yes

**********

**Reviewer #1:**  They addressed my comments before.

**Reviewer #2:**  There are still some non italicized pathogen names, eg lines 748-9, otherwise I am satisfied with the changes made to the manuscript.

**********

what does this mean?). If published, this will include your full peer review and any attached files.

**Do you want your identity to be public for this peer review?**  If you choose “no”, your identity will remain anonymous but your review may still be made public.

For information about this choice, including consent withdrawal, please see our Privacy Policy

Reviewer #1: No

Reviewer #2: **Yes:**  Jimstan Perisleneris

**********
